# Full-length transcriptome profiling reveals insight into the cold response of two kiwifruit genotypes (*A. arguta*) with contrasting freezing tolerances

**DOI:** 10.1186/s12870-021-03152-w

**Published:** 2021-08-11

**Authors:** Shihang Sun, Miaomiao Lin, Xiujuan Qi, Jinyong Chen, Hong Gu, Yunpeng Zhong, Leiming Sun, Abid Muhammad, Danfeng Bai, Chungen Hu, Jinbao Fang

**Affiliations:** 1 Key Laboratory for Fruit Tree Growth, Development and Quality Control, Zhengzhou Fruit Research Institute, Chinese Academy of Agricultural Sciences, Zhengzhou, 450009 China; 2 Key Laboratory of Horticultural Plant Biology (Ministry of Education), College of Horticulture and Forestry Science, Huazhong Agricultural University, Wuhan, 430070 China

**Keywords:** Low temperature, Kiwifruit, Freezing tolerance, Full-length transcriptome, Cold stress

## Abstract

**Background:**

Kiwifruit (*Actinidia* Lindl.) is considered an important fruit species worldwide. Due to its temperate origin, this species is highly vulnerable to freezing injury while under low-temperature stress. To obtain further knowledge of the mechanism underlying freezing tolerance, we carried out a hybrid transcriptome analysis of two *A. arguta* (*Actinidi arguta*) genotypes, KL and RB, whose freezing tolerance is high and low, respectively. Both genotypes were subjected to − 25 °C for 0 h, 1 h, and 4 h.

**Results:**

SMRT (single-molecule real-time) RNA-seq data were assembled using the de novo method, producing 24,306 unigenes with an N50 value of 1834 bp. Kyoto Encyclopedia of Genes and Genomes (KEGG) enrichment analysis of DEGs showed that they were involved in the ‘starch and sucrose metabolism’, the ‘mitogen-activated protein kinase (MAPK) signaling pathway’, the ‘phosphatidylinositol signaling system’, the ‘inositol phosphate metabolism’, and the ‘plant hormone signal transduction’. In particular, for ‘starch and sucrose metabolism’, we identified 3 key genes involved in cellulose degradation, trehalose synthesis, and starch degradation processes. Moreover, the activities of beta-GC (beta-glucosidase), TPS (trehalose-6-phosphate synthase), and BAM (beta-amylase), encoded by the abovementioned 3 key genes, were enhanced by cold stress. Three transcription factors (TFs) belonging to the AP2/ERF, bHLH (basic helix-loop-helix), and MYB families were involved in the low-temperature response. Furthermore, weighted gene coexpression network analysis (WGCNA) indicated that *beta-GC*, *TPS5*, and *BAM3.1* were the key genes involved in the cold response and were highly coexpressed together with the *CBF3*, *MYC2*, and *MYB44* genes.

**Conclusions:**

Cold stress led various changes in kiwifruit, the ‘phosphatidylinositol signaling system’, ‘inositol phosphate metabolism’, ‘MAPK signaling pathway’, ‘plant hormone signal transduction’, and ‘starch and sucrose metabolism’ processes were significantly affected by low temperature. Moreover, starch and sucrose metabolism may be the key pathway for tolerant kiwifruit to resist low temperature damages. These results increase our understanding of the complex mechanisms involved in the freezing tolerance of kiwifruit under cold stress and reveal a series of candidate genes for use in breeding new cultivars with enhanced freezing tolerance.

**Supplementary Information:**

The online version contains supplementary material available at 10.1186/s12870-021-03152-w.

## Background

Low temperature is one of the main abiotic stresses affecting horticultural plant development and growth and also restricts the geographical distribution of plants [1, 2]. Because plants are sessile organisms, low temperature can lead to decreased cell membrane fluidity and water potential and loss of protein function, resulting in enormous amounts of damage [3]. However, throughout their long-term evolutionary development, plants have evolved a set of complex mechanisms to adapt to low temperatures [4]. Freezing tolerance (FT) is considered an essential ability of plants to survive low-temperature stress and is an adaptive process during which the expression of proteins related to transcriptional regulation and functional materials is induced [5, 6]. In addition, complex metabolic regulatory activity is involved in FT, and metabolites such as antioxidant compounds (SOD, ascorbic acid), osmolytes (raffinose, trehalose) and nitrogenous compounds (glutathione and glycine betaine) have positive effects on increasing FT [7–9].

Genome data are basic information that can be used to facilitate research on FT mechanisms [10]. The *A. chinensis* (*Actinidia chinensis*) genome data were published in 2013; the genome has an ~ 140× sequencing depth, and the size of the current assembly is 616.1 Mb, with a total of 39,040 genes [11]. There are recently published kiwifruit genome assemblies for *A. chinensis* ‘Red 5’ and *Actinidia eriantha* ‘White’ as well as an improved assembly of ‘Hongyang’, all of which are diploid [12–14]. However, *A. arguta* is a tetraploid species, and its genome information is unknown due to genome complexity resulting from the polyploidy conditions. When genome data are absent, SMRT (single-molecule real-time) RNA-seq data can act as a useful tool to substitute for genomic [15]. SMRT long-read sequencing technology is currently becoming a popular method to obtain information on full-length (FL) mRNA molecules and has been extensively used for transcriptome profiling in various plant species [16–19]. FL transcript sequences that avoid incorrect assembly can show explicit information on each gene [20]. However, the SMRT method cannot calculate the mapped number of reads to quantify the expression level of genes, but this problem can be solved with NGS (next-generation sequencing) technology [21]. Furthermore, the SMRT sequencing method has a high error rate (up to 15%) for a single base, which can be corrected with NGS reads. Hence, hybrid RNA sequencing technology is a useful tool to obtain both qualitative and quantitative transcriptome results [22].

Transcriptome analysis has become an effective technology to characterize molecular regulatory activity and investigate the genes involved in response to various abiotic stresses, such as drought stress, waterlogging stress, and salt stress [23–25]. However, studies on transcriptional level changes under cold stress in kiwifruit are limited. In model plant species (*Arabidopsis*), previous studies have identified and confirmed several of the key genes and transcription factors (TFs) involved in the response to low-temperature stress [26]. Among these TFs, bHLH, MYB, and AP2/ERF TFs are considered important in terms of plant cold-responsive processes [27–29]. Many studies have shown the important roles of transcription factors, including CBFs (C-repeat binding factors), ICEs (inducers of CBF expression genes), and MYB15, in cold regulation and acclimation [30]. CBFs recognize and bind to *cis*-elements in the promoters of COR (cold-responsive) genes, thus triggering gene expression. At present, researchers have characterized several COR genes, including *ZAT*, *COR15a*, and *KIN* [31]. Moreover, metabolic pathways such as those of sugar metabolism, flavonoid metabolism, and amino acid metabolism are important in the response to cold stress [32–34]. Due to limited knowledge of the complex FT mechanism in kiwifruit, we planned to identify key pathways in kiwifruit through the transcriptome method.

*A. arguta*, unlike other *Actinidia* species, has an extensive geographic distribution varying from 25°N to 50°N. Moreover, these kiwifruit species have different FTs against cold stress. Especially in northern China, *A. arguta* has a remarkable FT, which implies that some changes in genetic mechanisms have occurred to enhance the FT of *A. arguta* [35]. However, studies on the FT mechanism in *A. arguta* are scarce. In this study, using SMRT sequencing and NGS analysis, we investigated two kiwifruit genotypes, KL and RB, with high and low FT, respectively. The physiological parameters of both genotypes were analyzed to strongly support the transcriptome analysis results. The present study aimed to identify differentially expressed genes and pathways involved in the FT of kiwifruit. Our results will reveal useful genes to develop new freezing-tolerant cultivars, which is important for coping with growing problems under low temperature for the kiwifruit industry in the near future.

## Results

### Physiochemical characteristics of kiwifruit under cold stress

**Fig. 1:**
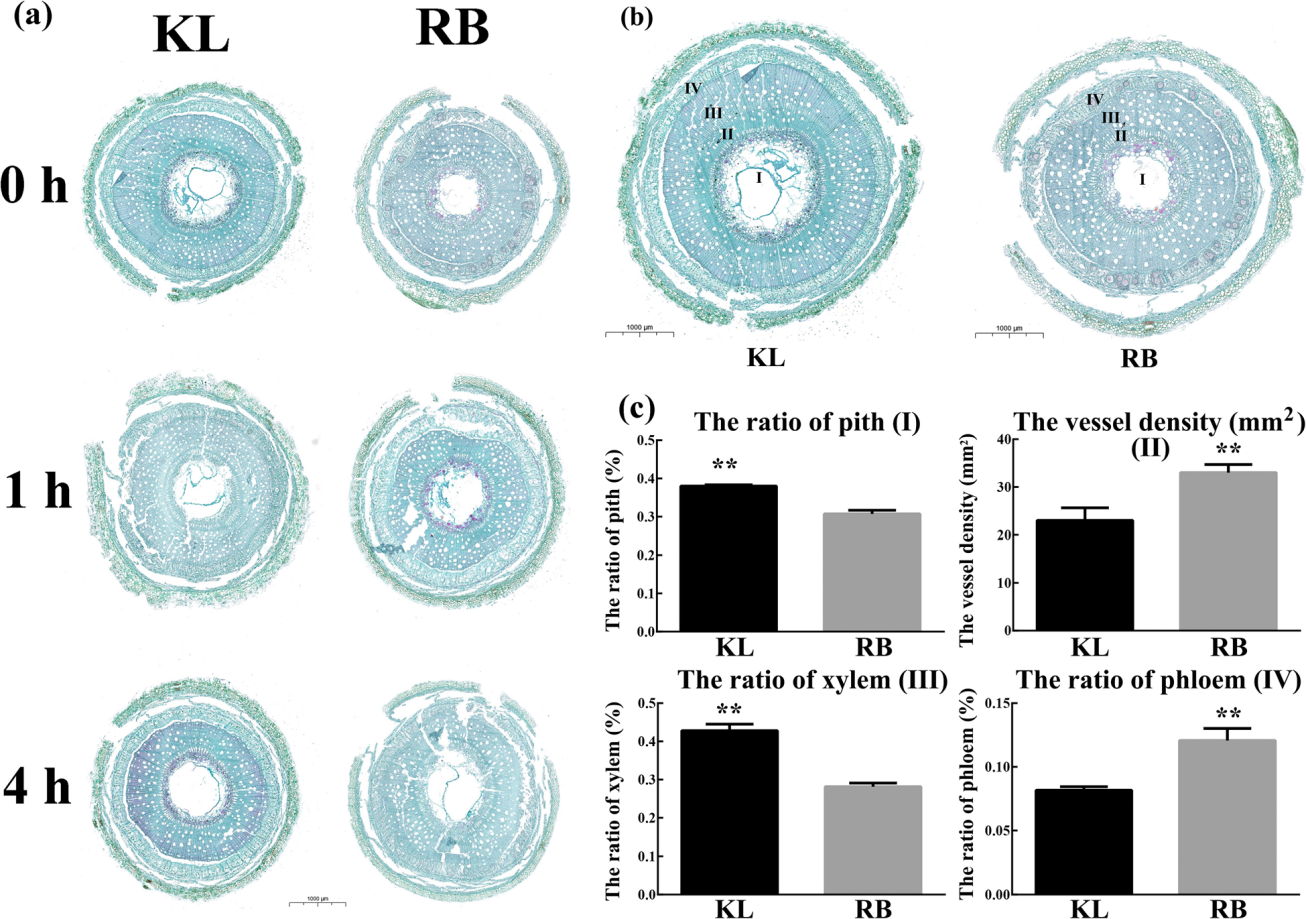
Microscopy observations of kiwifruit shoot anatomical structure. (a) Images of the anatomical structure of shoots after 0 h, 1 h, and 4 h of treatment (− 25 °C) for both genotypes. (b) Anatomical structure images of untreated shoots of both genotypes. I, the ratio of pith; II, the vessel density; III, the ratio of xylem; IV, the ratio of phloem. (c) Parameters of the anatomical structure of both genotypes. The bars represent the standard errors of the means (*n* = 3). The asterisks indicate that the values are significantly different (** for *P* < 0.01)

The shoots of two kiwifruit genotypes with contrasting freezing tolerance (KL: freezing-tolerant; RB: freezing-sensitive) were subjected to − 25 °C for 0 h, 1 h and 4 h. The anatomical structural changes of the shoots were observed under the microscope. The anatomical structure of the shoots was not significantly different under cold stress in either genotype (Fig. 1a). Due to the relationship between anatomical structure and freezing tolerance (FT), we further investigated several indices of both untreated genotypes. There were several differences in both genotypes, including in the ratio of pith, vessel density, ratio of xylem, and ratio of phloem. The ratio of pith and the ratio of xylem in KL were higher than those in RB, whereas the vessel density and ratio of phloem in RB were higher than those in KL (Fig. 1b, c). These differences suggested that geographical distribution led to anatomical structural changes in both genotypes.

**Fig. 2:**
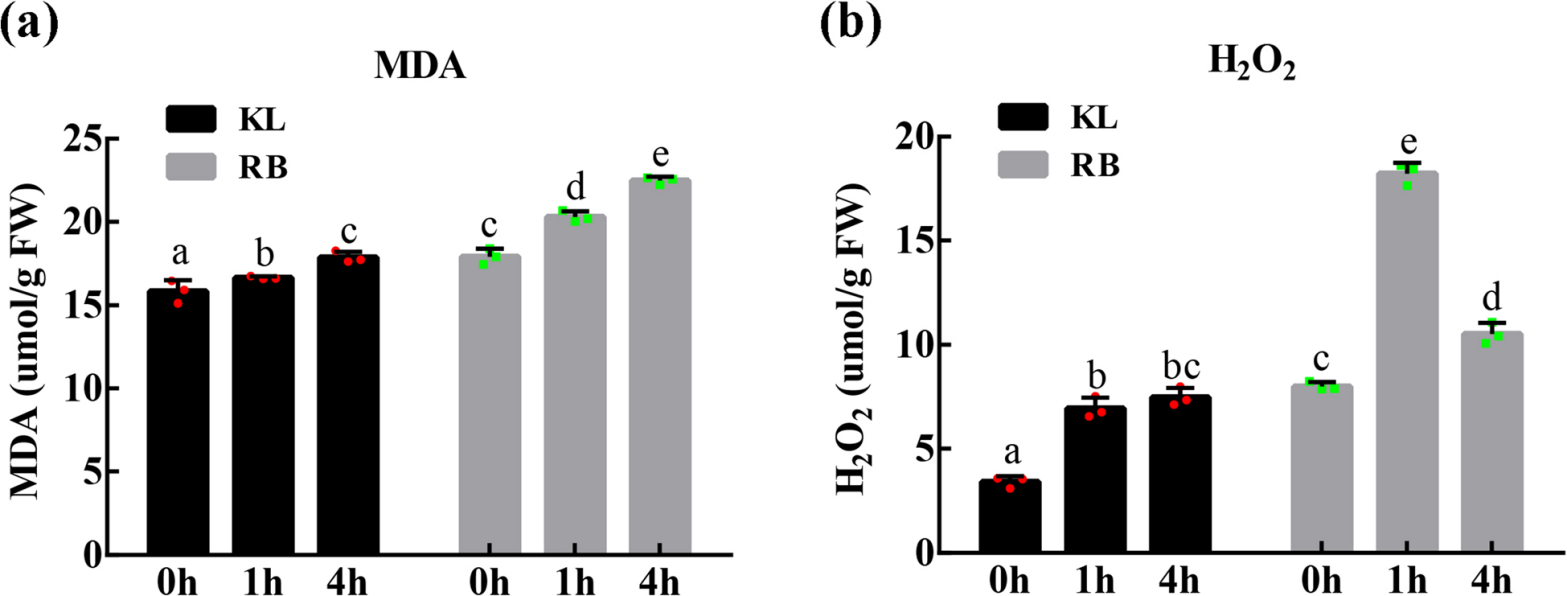
Comparative physiological characterization of kiwifruit shoots of two different genotypes. (a) Malondialdehyde (MDA) content in the shoots. (b) Hydrogen peroxide (H_2_O_2_) content in the shoots. The experiments included three biological replicates. Multiple comparisons were performed for significant differences (*P* < 0.05, two-way ANOVA and Tukey’s test; the error bars represent the SDs)

Two physiological indices, MDA content and H_2_O_2_ content, were measured to evaluate the FT of kiwifruit under cold stress. The plasma membrane has an important role in plants; however, low temperatures always lead to lipid peroxidation of the membrane. Membrane lipid peroxidation generates a set of metabolites, one of which is MDA. The MDA content in KL and RB showed a significant change from 0 h to 4 h (Fig. 2a). The MDA content in KL increased by approximately 13% from 0 h (15.8 μmol/g) to 4 h (17.9 μmol/g) at − 25 °C, whereas that in RB increased by approximately 26% from 0 h (17.9 μmol/g) to 4 h (22.5 μmol/g) at − 25 °C. Moreover, cold stress accelerated the accumulation of H_2_O_2_ in plants. The content of H_2_O_2_ in KL increased by 2-fold from 0 h (3.4 μmol/g) to 1 h (6.9 μmol/g) and then showed no significant change at 4 h (7.4 μmol/g); the content was consistent with that at 1 h. However, the H_2_O_2_ content in RB increased by 2.3-fold from 0 h (7.9 μmol/g) to 1 h (18.2 μmol/g) and then declined at 4 h (10.5 μmol/g) (Fig. 2b). Taken together, these results indicated that the membrane stability and level of oxidative stress in kiwifruit were affected by cold stress. Moreover, the damage that KL experienced from low-temperature stress was lower than that of RB.

### Summary of NGS and SMRT transcriptome sequencing of kiwifruit

**Fig. 3:**
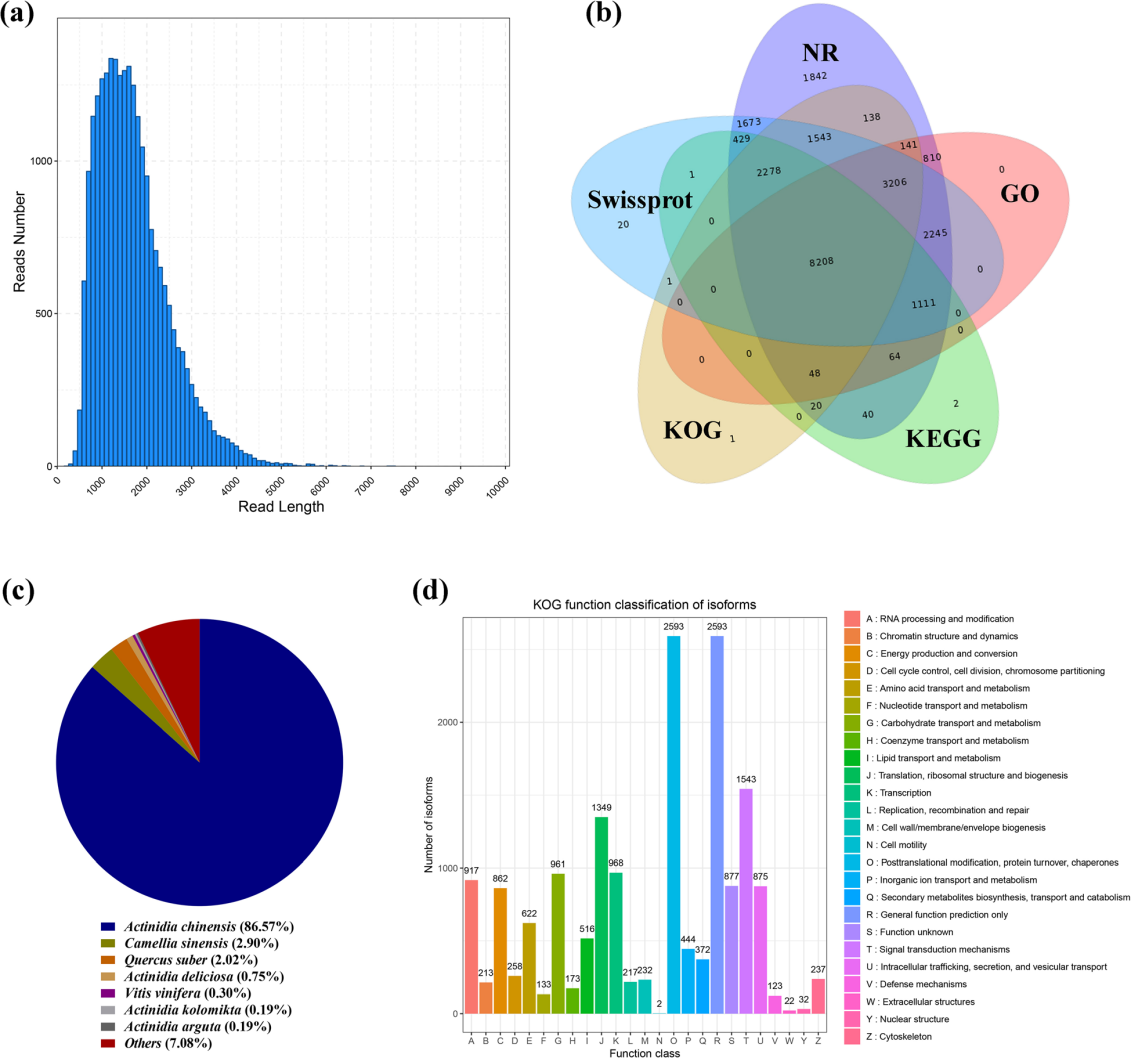
Overview of SMRT sequencing results and annotation of unigenes. (a) Distribution of full-length reads. (b) Overlap between the number of all unigenes according to five databases. (c) Distribution of unigene annotations based on the NR database for the species distribution statistics. (d) KOG functional classification of all unigenes

NGS transcriptome sequencing generated 906,672,934 raw reads and 866,664,476 clean reads (95.59%, 130.01 Gb) with an average Q30 value of 93.70% and an average GC content of 45.54% (Table 1). SMRT transcriptome sequencing generated 22,604,106 subreads (32.37 Gb in size, with an average length of 1388 bp and an N50 value of 1834 bp), that were corrected using the NGS data (Fig. 3a). A total of 350,711 circular consensus sequences (CCSs) were obtained from the filtration of the subreads. The number of full-length nonchimeric (FLNC) reads was 254,139, whose average length was 1602 bp.

**Table 1:** Summary of the transcriptome data from PacBio RSII platform and Illumina Hiseq platform

Platform	
**PacBio RSII**	
Total CCS	350711
Full length reads	271699
FLNC reads	254139
Average FLNC read length	1602 bp
Subreads base (G)	32.37 Gb
Subreads number	22604106
Average subreads length	1388
N50	1834
Total unigenes	24306
TF number	1953
**Illumina Hiseq**	
Raw reads (average)	50370718.56
Clean reads (average)	48148026.44
Clean base (G)	130.01 Gb
Base error rate (average)	0.03%
Q30 (average)	93.70%
GC content (average)	45.54%

### Functional annotation of unigenes

To determine the possible functions of unigenes in kiwifruit, a total of 24,306 unigenes in the SMRT transcriptome were functionally annotated using five databases (the NR, GO, KEGG, KOG, and SwissProt databases). All the unigenes had an approximately 98% annotation rate in at least one database [NR (23,796, 97.90%), GO (15,833, 65.14%), KEGG (12,201, 50.20%), KOG (15,584, 64.12%), and SwissProt (20,715, 85.23%)] (Fig. 3b).

To determine the conservation level of the unigene sequences of kiwifruit in other plant species, the unigene sequences in kiwifruit were queried in the NCBI NR database (Fig. 3c). The unigene sequences in kiwifruit displayed the highest similarity to sequences from *A. chinensis* (86.57%), followed by those from *Camellia sinensis* (2.90%), *Quercus suber* (2.02%), *Actinidia deliciosa* (0.75%), *Vitis vinifera* (0.30%), *Actinidia kolomikta* (0.19%), and *A. arguta* (0.19%). The results suggested that the majority of unigenes were annotated in *A. chinensis*.

All the kiwifruit unigenes were subsequently queried against the KOG database (Fig. 3d). A total of 15,584 sequences were annotated, with 26 functional categories. ‘Posttranslational modification, protein turnover, and chaperones’ (2593, 16.64%); ‘general function prediction only’ (2593, 16.64%); and ‘signal transduction mechanisms’ (1543, 9.90%) were the top three categories. Furthermore, ‘cell motility’ (2, 0.012%) was the smallest category with the fewest unigenes.

A total of 15,833 unigenes were annotated in the GO database (Fig. S1), which were successfully clustered into 48 functional groups, of which 14 categories belonged to BPs (biological processes), 22 belonged to CCs (cellular components), and 12 belonged to MFs (molecular functions). We also conducted an analysis based on KEGG pathways to obtain key information, including that on intracellular metabolic pathways and biological functions of genes in kiwifruit (Fig. S2). A total of 12,201 unigene sequences were clustered into 19 KEGG pathway categories. Moreover, the most significant category among these pathways was ‘carbohydrate metabolism’ (1446; 11.85%), followed by ‘translation’ (1406; 11.52%) and then ‘folding, sorting and degradation’ (1310; 10.73%).

### Identification of DEGs

**Fig. 4:**
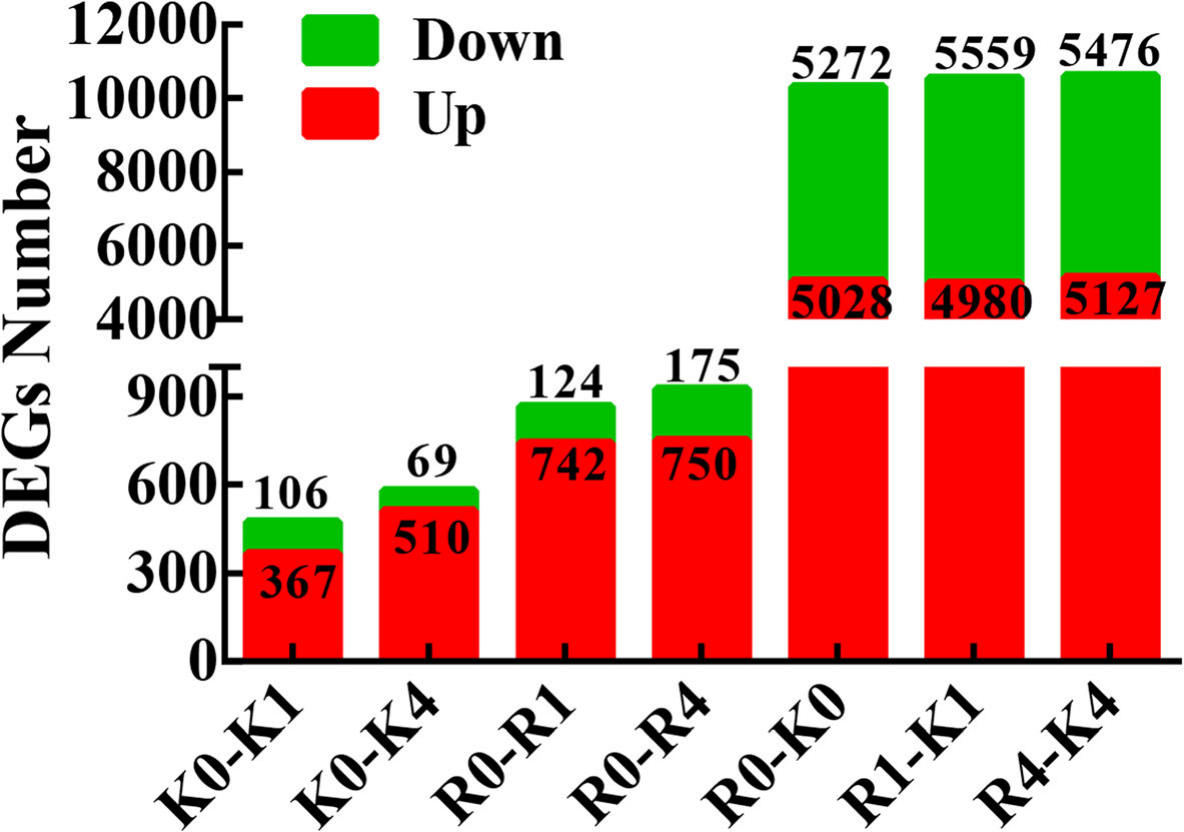
Summary of differential expression analysis of kiwifruit under cold stress. The numbers of upregulated and downregulated DEGs were identified in each comparison of different time intervals. K0, KL-0 h; K1, KL-1 h; K4, KL-4 h; R0, RB-0 h; R1, RB-1 h; R4, RB-4 h

DEGs in the two contrasting genotypes were screened from a combination of NGS transcriptome data and SMRT transcriptome data. The expression levels of DEGs were compared between every two groups based on the FPKM values (Fig. 4). There were 473 DEGs (367 up- and 106 downregulated) between KL-0 h and KL-1 h, 579 DEGs (510 up- and 69 downregulated) between KL-0 h and KL-4 h, 866 DEGs (742 up- and 124 downregulated) between RB-0 h and RB-1 h, 925 DEGs (750 up- and 175 downregulated) between RB-0 h and RB-4 h, 10,300 DEGs (5028 up- and 5272 downregulated) between RB-0 h and KL-0 h, 10,539 DEGs (4980 up- and 5559 downregulated) between RB-1 h and KL-1 h, and 10,603 DEGs (5127 up- and 5476 downregulated) between RB-4 h and KL-4 h. These results indicated that the number of DEGs increased after low-temperature treatment in both genotypes. Moreover, RB exhibited more DEGs than did KL at 1 h and 4 h of treatment. For example, the number of DEGs in RB after 1 h of treatment was 866, which was almost twice that in KL DEGs (473). These differences in DEG numbers indicated that cold stress could induce more dynamic transcriptomic changes in RB than in KL and that KL had a stronger ability to maintain stable transcriptomes under cold stress. Furthermore, among the 3 time points, 4 h was the specific treatment time point that had the most abundant DEGs.

### KEGG classification of DEGs

**Fig. 5:**
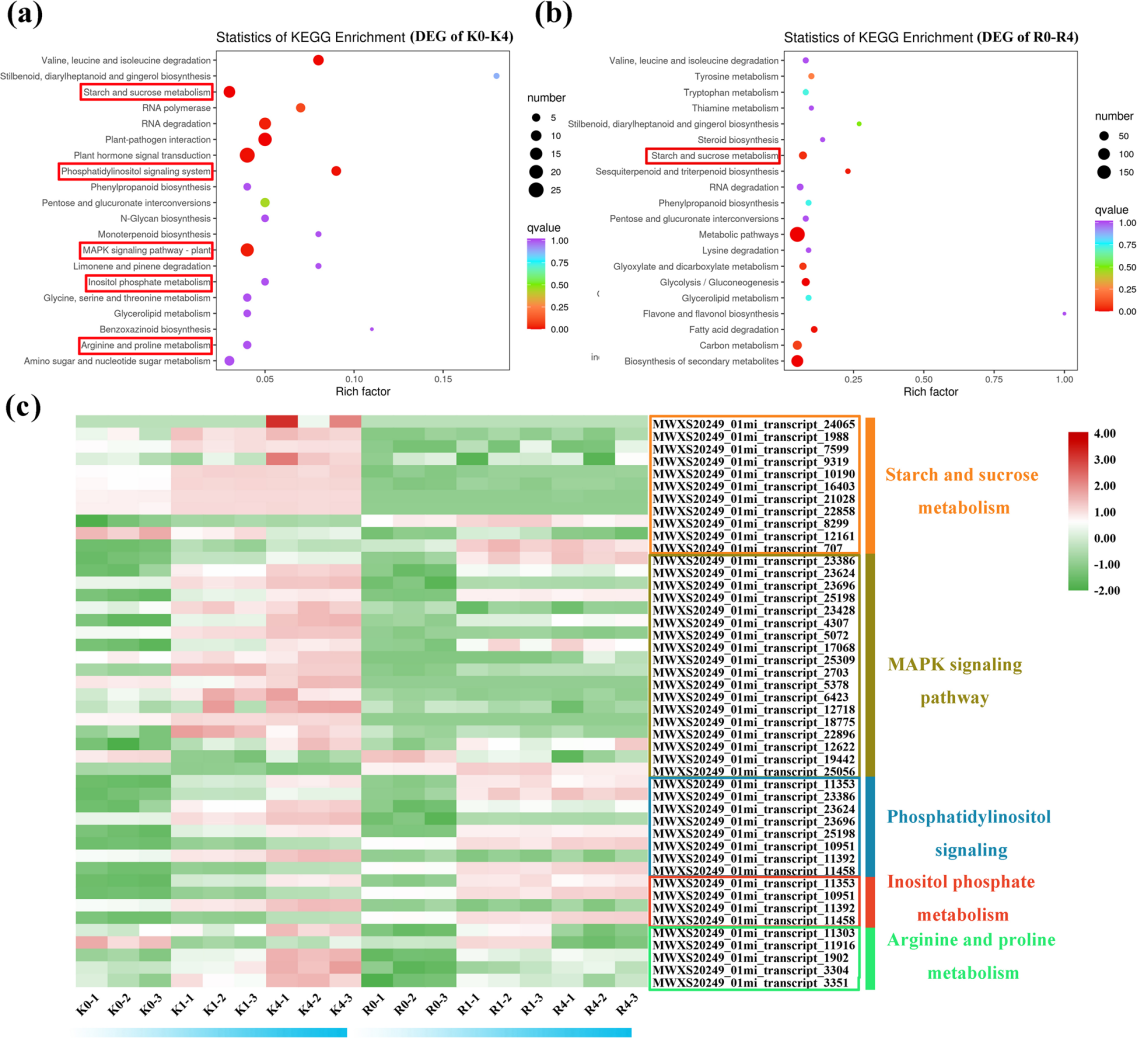
KEGG enrichment of DEGs identified at 0 h vs. 4 h. (a) KEGG enrichment of DEGs between K0 (KL-0 h) and K4 (KL-4 h). The red boxes represent key pathways involved in the response to cold stress. (b) KEGG enrichment of DEGs between R0 (RB-0 h) and R4 (RB-4 h). The red boxes represent key pathways involved in the response to cold stress. (c) Heat map constructed based on the FPKM value of each gene of the key pathways

The DEGs identified in KL between 0 h and 4 h were queried in the KEGG pathway database (Fig. 5a). KEGG pathway enrichment showed that the most significant subcategory among a series of pathways was ‘starch and sucrose metabolism’, followed by the ‘MAPK signaling pathway’, the ‘phosphatidylinositol signaling system’, ‘inositol phosphate metabolism’, and ‘arginine and proline metabolism’ in KL. However, KEGG analysis of RB between 0 h and 4 h showed that ‘starch and sucrose metabolism’ was involved in the response to cold stress (Fig. 5b). For the ‘starch and sucrose metabolism’ pathway, 11 transcripts were involved in this pathway, while most (9 out of 11) transcripts continuously had higher expression in KL than in RB. For the ‘MAPK signaling pathway’, 13 out of 18 transcripts involved in the MAPK signaling pathway had higher expression in KL than in RB. For the ‘phosphatidylinositol signaling system’ pathway, 5 of 8 transcripts had higher expression in KL than in RB. For the ‘inositol phosphate metabolism’ pathway, 2 transcripts of 4 transcripts had higher expression in KL, and for the ‘arginine and proline metabolism’ pathway, 4 of 5 transcripts had higher expression in KL (Table S1) (Fig. 5c). Taken together, these results highlighted the involvement of different pathways in the response to low-temperature stress.

### Identification of DEGs related to phytohormones and signaling

**Fig. 6:**
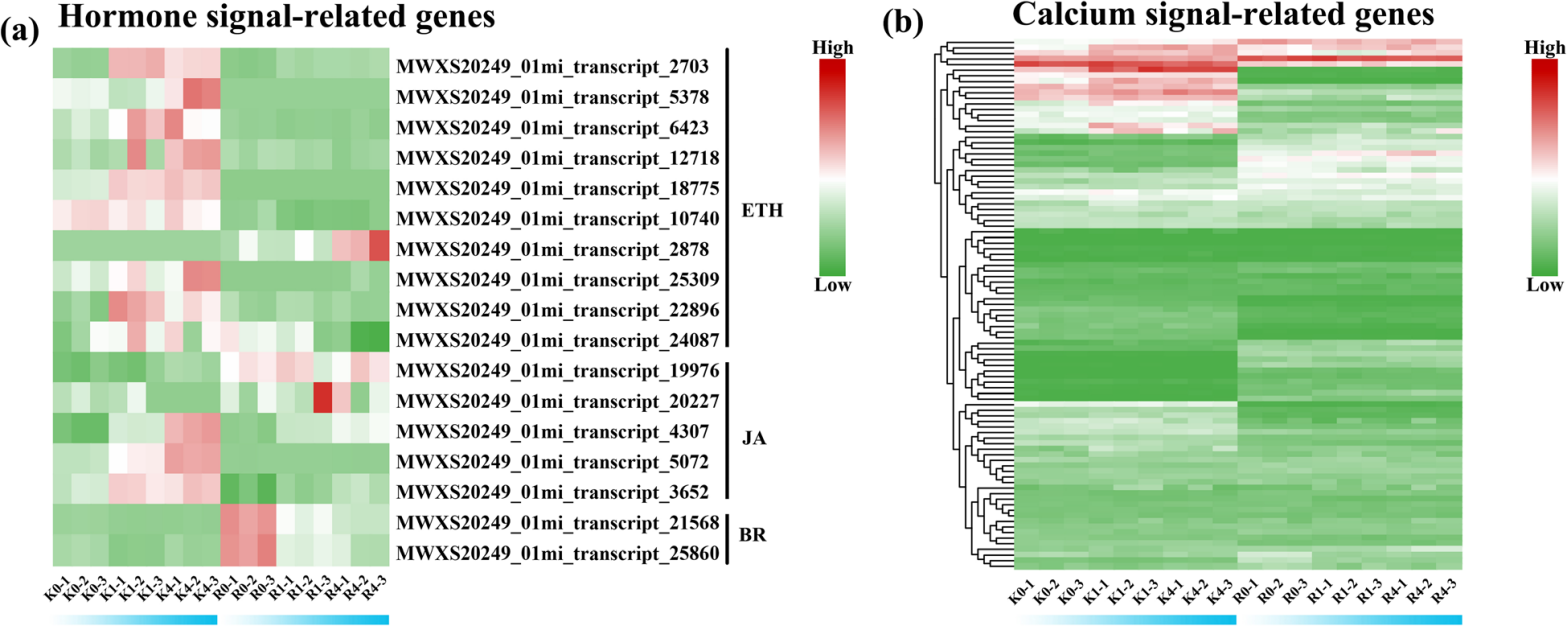
Heat map of signal transduction-related genes in cold-stressed kiwifruit. (a) Heat map constructed based on hormone signal-related genes. (b) Heat map constructed based on calcium signal-related genes. Ethylene, ETH; jasmonic acid, JA; brassinolide, BR

Among the DEGs, we identified several that were associated with phytohormones and calcium signaling. Seventeen DEGs involved in phytohormone pathways were selected, and their expression patterns are illustrated in Fig. 6a; these DEGs included 10 genes involved in the ethylene signaling process, 5 genes involved in the jasmonic acid signaling process, and 2 genes involved in the brassinosteroid signaling process (Table S2). For the ethylene process, 9 out of 10 genes were induced by cold stress in KL; these genes were expressed at levels higher than those in RB. The abovementioned 9 genes encoded ERF, EIN, and EBF proteins. For the jasmonic acid signaling process, 3 out of 5 genes had higher expression in KL than in RB; the above 3 genes encoded MYC2. For the brassinosteroid process, both genes had different expression levels in the two genotypes, and there were no changes in KL, whereas cold stress decreased the expression of 2 genes encoding TCH4 (xyloglucan endotransglucosylase) in RB. Moreover, a total of 96 transcripts encoded calcium signaling-related genes, including 1 CRLK (calcium/calmodulin-regulated receptor-like kinase), 14 CAMTAs (calmodulin-binding transcription activators), 22 CIPKs (CBL-interacting protein kinases), 46 CDPKs (calcium-dependent protein kinases), and 12 CNGCs (cyclic nucleotide/calmodulin-gated ion channels). Seventeen genes were significantly induced by cold stress in KL; however, 5 genes were significantly induced by low temperature in RB; the 12 genes with specific expression in KL included 1 CAMTA, 5 CIPK genes, 2 CDPK genes, and 4 CNGC genes (Fig. 6b).

### Identification of DEGs related to starch and sucrose metabolism and measurement of sugar metabolites

**Fig. 7:**
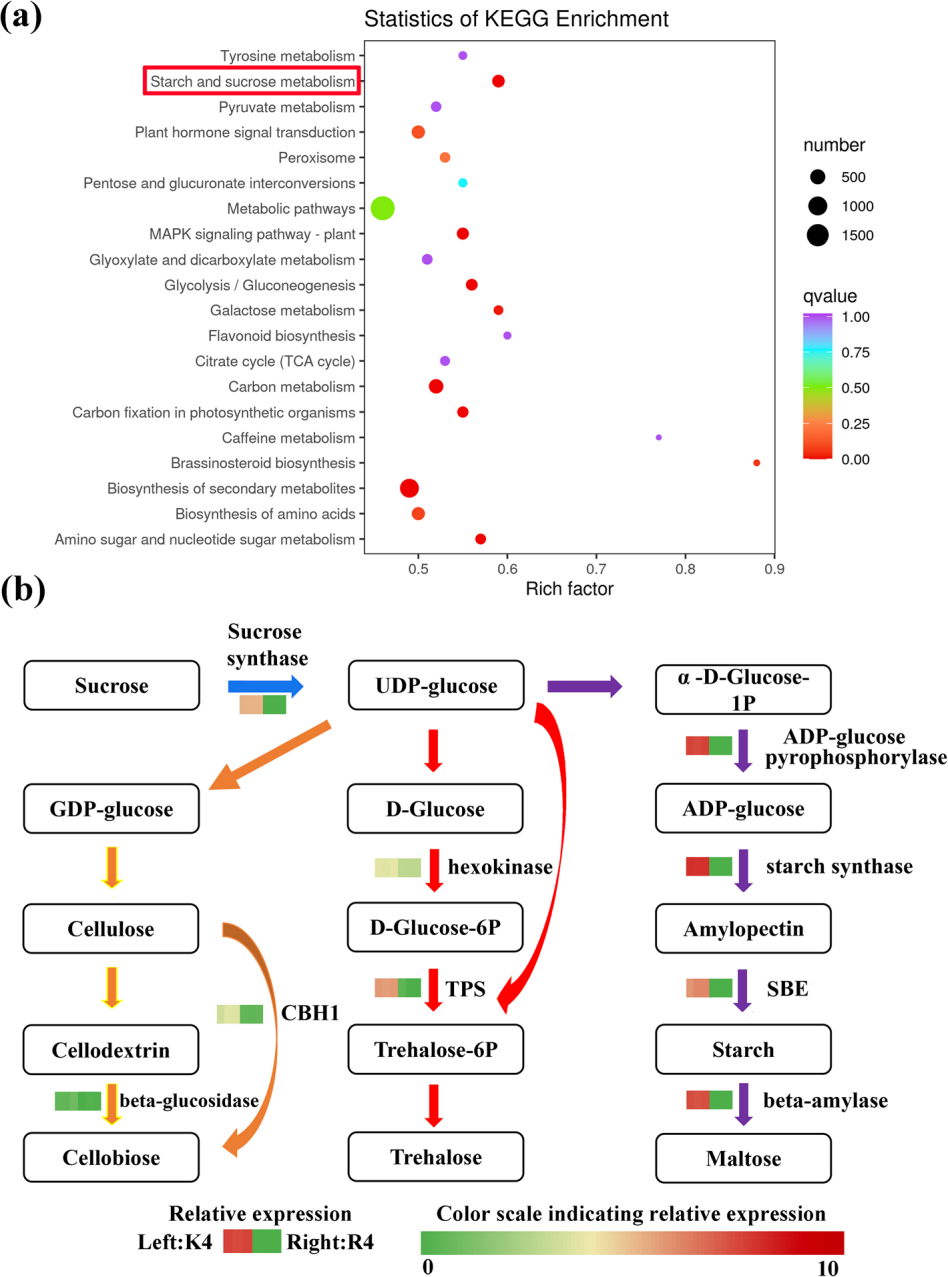
(a) KEGG enrichment of DEGs between KL-4 h and RB-4 h. The red box represents the key pathway involved in the response to cold stress. (b) DEGs involved in the starch and sucrose metabolism pathways. Gene expression is represented by the grids under the 4 h treatment, with FPKM values of both genotypes shown

A comparison between the KL-4 h and RB-4 h groups was performed. A KEGG enrichment scatterplot of the DEGs was constructed to provide a graphical presentation of the KEGG enrichment analysis results (Fig. 7a). The ‘starch and sucrose metabolism’ pathway, which was strongly enriched, was selected and presented. There were 3 significant metabolic processes in ‘starch and sucrose metabolism’: ‘cellulose degradation’, ‘trehalose synthesis’, and ‘starch degradation’. Nine DEGs were involved in the above 3 significant metabolic processes. Among these 9 functional genes, those encoding SS (sucrose synthase), CBH1 (cellobiohydrolase), beta-glucosidase (beta-GC), TPS, hexokinase, ADP-glucose pyrophosphorylase, SS (starch synthase), SBE (starch branching enzyme), and beta-amylase (BAM) were highly expressed in KL compared with RB (Fig. 7b). Therefore, we speculated that starch degradation, trehalose synthesis, and cellulose degradation played an important role in enhancing FT.

**Fig. 8:**
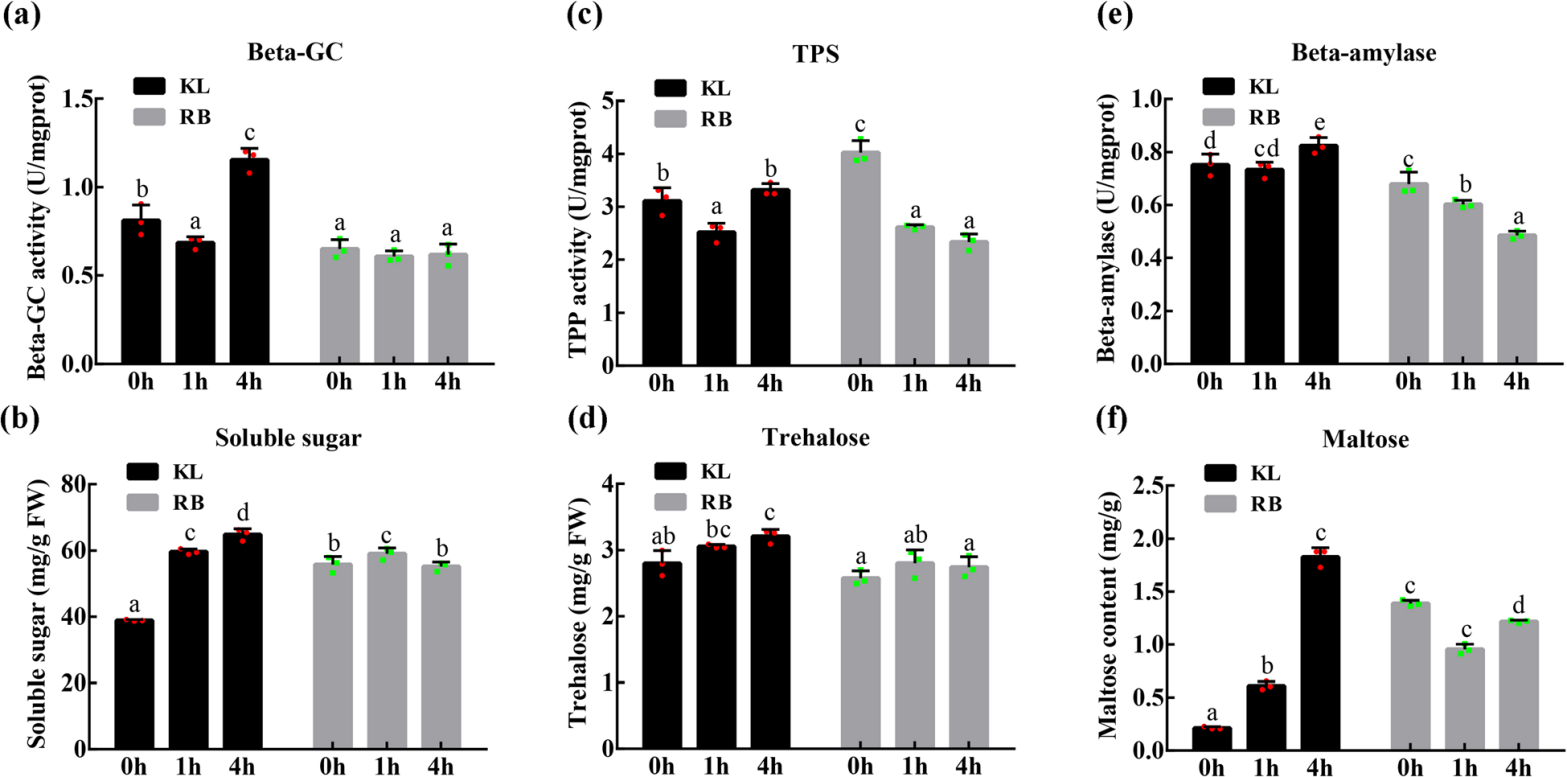
Changes in beta-GC (a), soluble sugar (b), TPS (c), trehalose (d), beta-amylase (e), and maltose (f) contents in the shoots of both genotypes under cold stress

The beta-GC, TPS, and BAM enzymes are key enzymes involved in ‘cellulose degradation’, ‘trehalose synthesis’, and ‘starch degradation’ processes, respectively. These enzymes were affected by cold stress but showed different changes in activity in both genotypes under cold stress (Fig. 8a, c, e). Under cold stress, beta-GC activity in KL showed an initial decrease and then an increase. However, in RB, beta-GC activity showed no significant change. Cold stress caused an increase in TPS activity. In KL, TPS activity remained relatively high; in contrast, cold stress suppressed TPS activity in the cold period in RB. BAM activity increased under cold stress in KL, whereas it decreased in RB. In addition, the BAM activity in KL was higher than that in RB throughout the measured period.

The level of soluble sugar in kiwifruit shoots was also measured. Cold stress increased the soluble sugar content throughout the cold treatment in KL, and there was no significant difference in the soluble sugar content between the 4 h and 0 h treatments in RB (Fig. 8b). As shown in Fig. 8d, KL showed increased trehalose content at 4 h, whereas RB showed no significant difference throughout the cold treatment. Under cold stress, cold increased the maltose content in KL but decreased it in RB (Fig. 8f). These results indicated that kiwifruit actively stimulated a set of starch metabolic pathways to enhance FT to resist freezing damage during the low-temperature treatment periods. In addition, low temperature may promote soluble sugar, trehalose and maltose accumulation under cold stress to increase FT.

### TFs in response to low-temperature stress

**Fig. 9:**
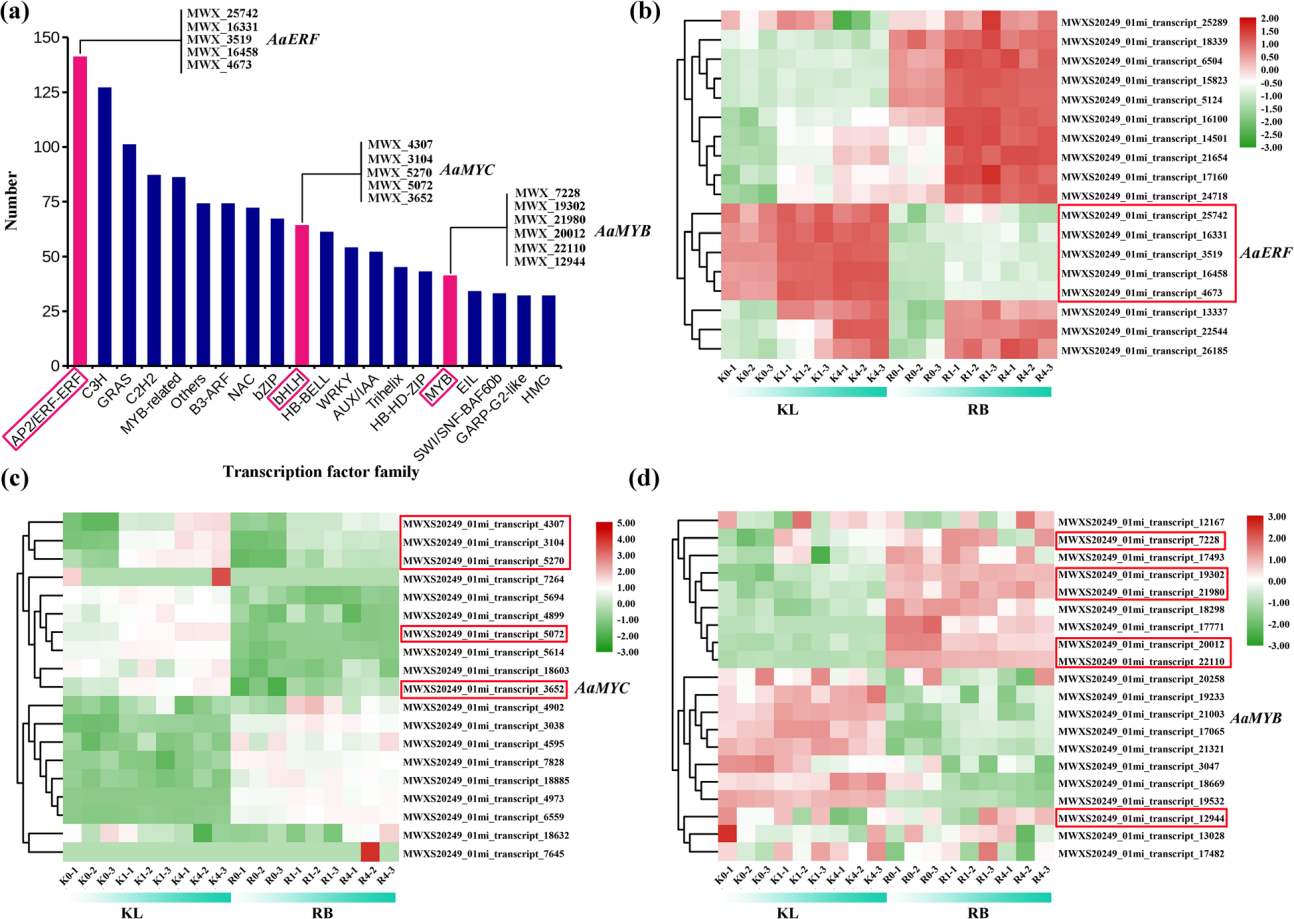
Number and classification of TF-encoded genes among the unigenes (a). Heat map constructed based on FPKM expression values of ERFs (b), MYCs (c), and MYBs (d)

Transcription factors play a key role in cold stress and are involved in transcriptional regulatory networks. A total of 20 categories of TF families were identified (Fig. 9a). The DEGs encoding most of the TFs were involved in the low-temperature response, and the majority belonged to the ERF, bHLH, and MYB families. Members of 3 kinds of TF families have been previously identified to regulate FT. Consequently, DEGs derived from three transcription factor families (ERF, bHLH, and MYB) are shown in Fig. 9b, c, d. The 16 TFs (Table S3) were screened, 5 of which belonged to the ERF, 5 belonged to the bHLH, and 6 belonged to the MYB families. The 5 *AaERF* genes and 5 *AaMYC* genes had higher expression in KL than in RB, and 6 *AaMYB* genes had lower expression in KL than in RB. In addition to the abovementioned ERF, bHLH, and MYB family members, 4 genes belonging to the C2H2 family encode zinc finger proteins (ZAT6, ZAT10, and ZAT12), which had higher expression in KL than in RB; other members of TFs, including 1 HSF gene, 1 NAC gene, and 1 WRKY gene, were also significantly increased upon cold stress (Table 2).

**Table 2:** FPKM values of differentially expressed TF in both genotypes

ID	Name	K0	K1	K4	R0	R1	R4
MWXS20249_01mi_transcript_23086	ZAT6	27.10	127.74	169.98	6.75	15.77	17.10
MWXS20249_01mi_transcript_23199	ZAT10	11.69	97.55	162.67	3.30	68.27	62.87
MWXS20249_01mi_transcript_24340	ZAT10	61.80	259.50	362.35	44.81	181.13	163.13
MWXS20249_01mi_transcript_25307	ZAT12	0.81	14.02	30.38	0.00	0.00	0.00
MWXS20249_01mi_transcript_2738	HSF	4.87	11.46	19.73	3.87	13.35	11.02
MWXS20249_01mi_transcript_8860	NAC	2.97	14.45	15.23	1.27	3.78	3.33
MWXS20249_01mi_transcript_4511	WRKY	9.24	19.70	29.89	6.20	12.51	14.13

### Construction of WGCNA networks and identification of candidate genes in kiwifruit

**Fig. 10:**
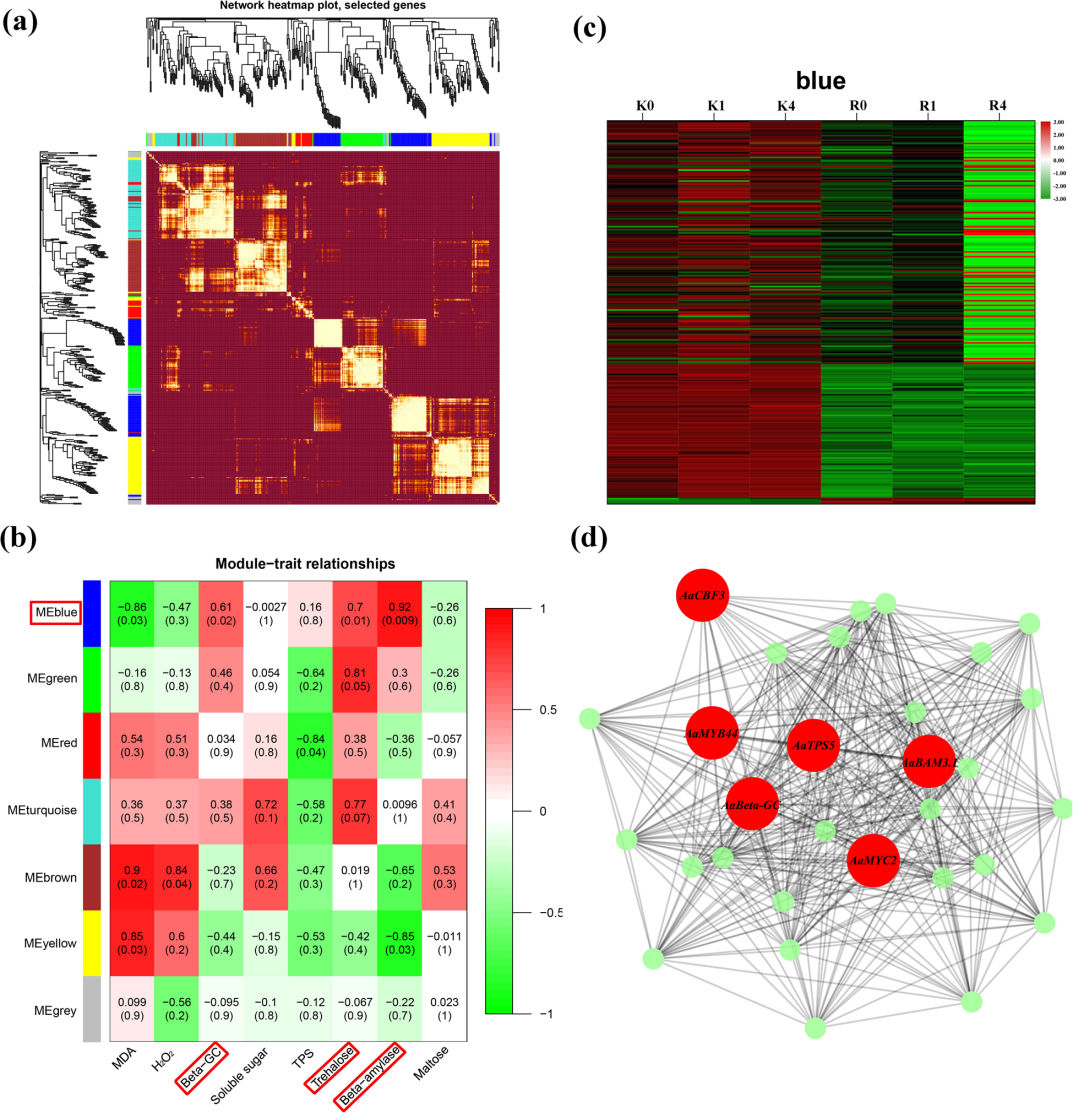
Identification of coexpression network modules in kiwifruit. (a) Cluster dendrogram and heat map of genes subjected to coexpression module calculations. (b) Module-trait associations based on Pearson correlations. The color key from green to red represents *r*^*2*^ values from − 1 to 1, respectively. (c) Heat map constructed for the blue module genes. (d) Gene network of the blue module, which is positively correlated with beta-GC (*r*^*2*^ = 0.61), trehalose (*r*^*2*^ = 0.7), and BAM (*r*^*2*^ = 0.92) contents. The candidate genes within the blue module are highlighted in red due to the higher weight within the module and coded for gene descriptors based on annotations

To determine the correlation between samples and modules, we constructed a gene coexpression network using RNA-seq data. A network heatmap (interconnectivity plot) of the gene network is shown, which displays the resulting modules and corresponding hierarchical clustering dendrograms (Fig. 10a). Seven modules containing 60–251 DEGs each were identified. MDA, H_2_O_2_, beta-GC, soluble sugar, TPS, trehalose, BAM, and maltose contents were used as phenotypic data for calculating the relationships in the trait modules. The results of the correlation between the modules and starch-sucrose pathway indicated that the correlation values ranged from − 0.85 to 0.92 (Fig. 10b). The eigengenes of the blue modules showed significant correlations with the starch-sugar pathway. The heat map of the blue module showed that FPKM values were higher in KL than in RB (Fig. 10c). To further identify key candidate genes in the blue module, the hub genes in the blue module were calculated using Cytoscape software (Table S4). The results of the hub gene search showed that the connections of the sugar-related and transcription factor-regulated DEGs were in the blue module, and sugar-related genes were highly coexpressed with transcription factor-regulated genes (Fig. 10d).

### Validation of candidate genes by qRT-PCR

**Fig. 11:**
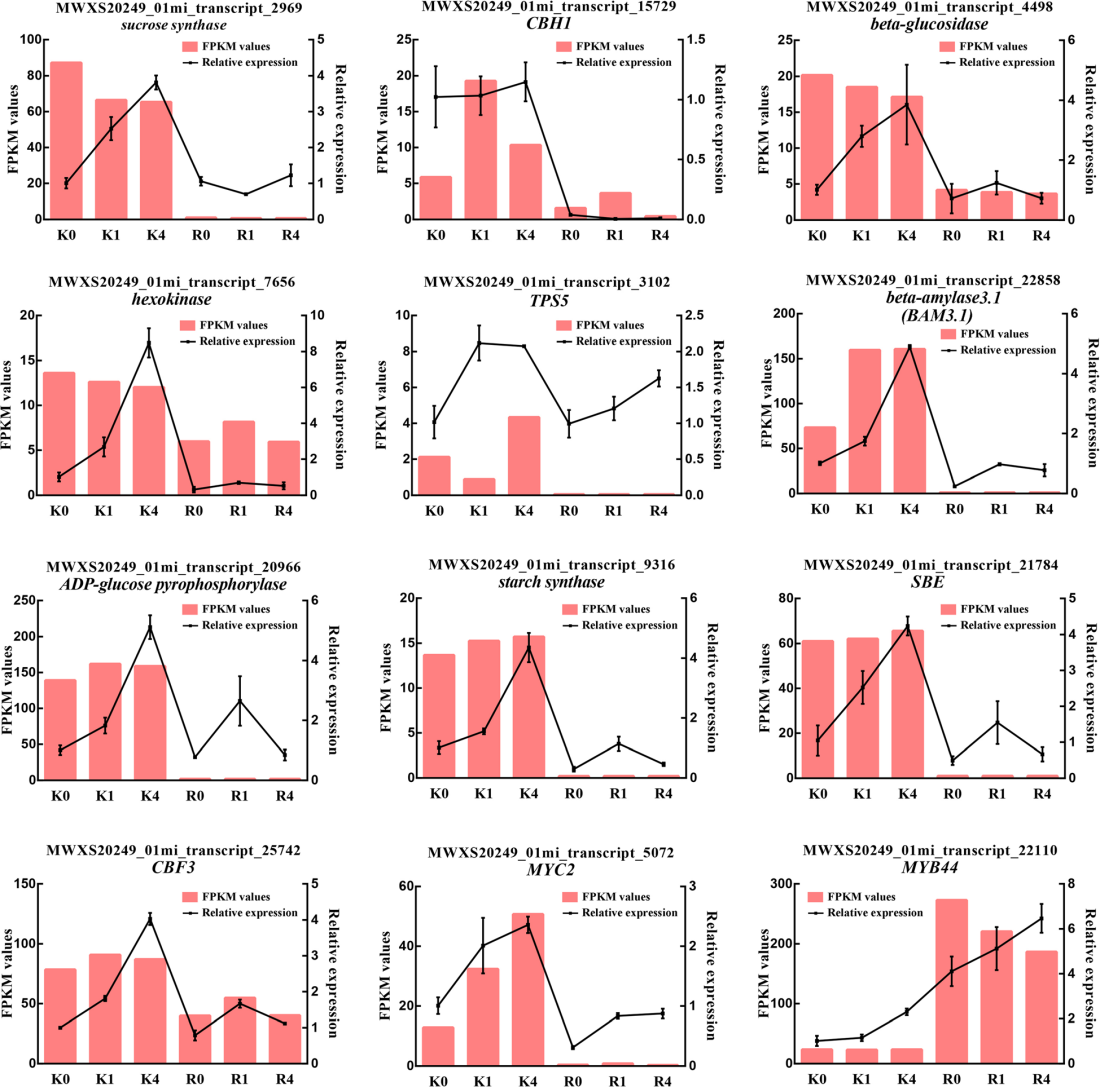
qRT-PCR verification of the expression pattern according to the RNA-seq data. The relative expression levels were calculated according to the 2^-∆∆CT^ method, with the actin reference gene serving as a control

For further validation of the RNA-seq results and candidate genes linked to starch and sucrose metabolism, we selected 9 DEGs involved in ‘starch and sucrose metabolism’ and 3 DEGs identified as potential candidate TFs for qRT-PCR experiments. While the fold-change of gene expression levels measured by qRT-PCR and RNA-seq were different, the expression patterns of the majority of the 12 DEGs assessed by the two methods showed similar trends, which validated the reliability of the RNA-seq results (Fig. 11). Moreover, these results confirmed that *beta-GC*, *TPS5*, *BAM3.1*, *CBF3*, *MYC2*, and *MYB44* were potential candidate genes involved in the regulation of FT (Fig. S3).

## Discussion

Kiwifruit is not only an important fruit species but also an economically important plant species worldwide. Cold stress is an abiotic stress that harms the development of kiwifruit and limits its yield. To enhance FT, it is essential to explore the FT mechanism of kiwifruit. However, the ploidy of kiwifruit is comparatively complex and leads to complex genomic information, and the breeding of cultivars with high FT is obstructed by the absence of functional genomic data. We therefore used a hybrid sequencing method combining both SMRT and NGS sequencing to determine the regulatory network of gene expression and induction in shoots of kiwifruit under cold stress. Moreover, physiological and transcriptome analyses were carried out to determine the FT responses in kiwifruit in this study.

### Involvement of starch and sucrose metabolism in high freezing tolerance

The enzymes and genes participating in starch degradation play an important role in the response to cold stress [36, 37]. In our study, a set of key genes associated with starch and sucrose metabolism were screened, and their expression levels were determined under cold stress. A majority of the identified genes were induced under cold stress, showing that cellulose degradation, trehalose accumulation and starch degradation were activated during this period, which may be important for the improvement of FT of kiwifruit (Fig. 8). Beta-glucosidase is a rate-limiting enzyme involved in the hydrolysis of cellulose [38]. Some beta-glucosidases can affect the structure of the cell wall, which might play a key role in cell adaptations to the physical deformations caused by cold stress [39]. After cold acclimation, *A. thaliana* plants with a mutation in sfr2–1, which encodes beta-glucosidase, are more sensitive to FT than the wild type is [40]. Under various abiotic stresses, beta-glucosidases hydrolyze inert precursors to release antioxidant substances [41]. A study on the transcriptional analyses of chickpea showed that the beta-glucosidase gene was screened to act as a differentially expressed gene under cold stress and that its expression was induced in response to low temperature [42]. Beta-glucosidase BGLU10 may be involved in cold, salt, and drought stress in *A. thaliana* [43]. In our study, the expression of the beta-glucosidase gene was upregulated under cold stress, and the expression of the beta-glucosidase gene in KL was higher than that in RB. Moreover, beta-glucosidase activity was activated, and the soluble sugar content also increased under cold stress.

Additionally, trehalose, a multifunctional disaccharide, exists extensively in various plant species [44, 45]. Trehalose forms a special protective structure on the surface of the cell membrane, protects the activity of functional protein molecules, and thus resists multiple abiotic stresses [46]. Both trehalose-6-phosphate and trehalose-phosphate-phosphatase play an important role in the synthesis of trehalose. Rice plants overexpressing *OsTPS1* and *OsTPP1* can acquire higher FT [47, 48]. TPS is the crucial controller, and there are 11 copies of the TPS gene in *A. thaliana*. Transgenic plants of *A. thaliana* overexpressing *TPP* and *TPS* increased the tolerance ability under low-temperature and salt stress [49]. Exogenous trehalose can induce the expression of stress-related genes in plants [50]. In this study, *TPS* genes were isolated and upregulated under cold stress. Moreover, OsICE1 can serve as a positively regulated TF to activate the expression of *OsTPP* [51]; likewise, we identified that both *MYC2* and *TPS* were induced under cold stress in KL.

BAM is an important hydrolase that degrades starch to yield maltose and has a key effect on FT improvement [52]. The expression and activity of BAM were reported to be significantly induced after a 6 h treatment of cold stress in *A. thaliana* [53]. In kiwifruit, *AaBAM3* was upregulated to increase the activity of beta-amylase from autumn to winter in China [54]. In pear, researchers have found the same results as those in kiwifruit [55]. Interestingly, in *Poncirus trifoliata*, there is a CBF-binding element in the promoter of PtrBAM1, and CBF can regulate the expression of PtrBAM1, which plays a role in FT by regulating the sugar metabolite content [56]. In our study, the results of kiwifruit were similar to those of other plant species, and *BAM* was upregulated under cold stress. In general, the abovementioned 3 genes may be key genes that enhance the FT of KL.

### Pathways involved in cold signaling

The phosphatidylinositol signaling system and the inositol phosphate metabolic pathways have been reported to be associated with FT [57, 58]. The significant KEGG pathways during the 4 h treatment were enriched in both of these pathways in KL but not in RB (Fig. 5a, b). These results of the KEGG analyses showed that the gene expression levels of the different genotypes were different under low-temperature stress. Inositol 3-phosphate (IP3), which is generated by phospholipase C-mediated hydrolysis of phosphatidylinositol bisphosphate (PIP2), triggers a set of cellular processes by releasing calcium. These releases can increase calcium from low to high levels. Calcium also acts as a secondary messenger to transduce cold signals. In *A. thaliana*, the phosphatidylinositol-hydrolyzing phospholipase gene was upregulated under multiple abiotic stresses [59, 60]. Moreover, myo-inositol-3-phosphate synthase activity was shown to be activated during the ABA signaling response in *Spirodela polyrhiza* [61]. Overexpression of D-myo-inositol-3-phosphate synthase leads to elevated levels of inositol in *Arabidopsis* under salt stress [62]. In our study, three transcripts (MWXS20249_01mi_transcript_10951, MWXS20249_01mi_transcript_11392, MWXS20249_01mi_transcript_11458) encoding myotubularin phosphatases were identified; these proteins subject phosphatidylinositol to different phosphorylation patterns, resulting in the production of distinct phosphatidylinositol phosphates. The genes encoding myotubularin phosphatases were induced under cold stress to increase FT. Inositol-1,3,4-trisphosphate 5/6-kinase (MWXS20249_01mi_transcript_11353) is involved in inositol phosphate metabolism, which can also trigger signal transduction. Hence, KL might enhance its FT by activating genes related to both the phosphatidylinositol signaling system and the inositol phosphate metabolic pathways.

Plant hormones play an important role in regulating stress responses and activating downstream low-temperature pathways. ETH, JA, and BR hormones are vital stress hormones involved in the modulation of the responses to cold stress. We noticed that several ETH signaling-related genes, such as ERF, EIN, and EBF, were upregulated during cold stress in the freezing-tolerant genotype (KL). Both the stress-responsive JA signaling-related genes *JAZ* and *MYC* were induced under cold stress in KL (Fig. 6a); relatedly, PtrMYC2 of *Poncirus trifoliata* integrates JA signals to modulate cold-induced GB accumulation by directly regulating the expression of PtrBADH-l, a betaine aldehyde dehydrogenase (BADH)-like gene [9]. BR signaling is also the key pathway controlling the response to cold stress, and brassinazole-resistant 1 (BZR1) was shown to be upregulated under cold stress in *Poncirus trifoliata* to enhance its FT [63]. Furthermore, the TCH4 gene acts downstream of BZR1 [64]. In our study, we identified two TCH4 genes, and these genes were repressed under cold stress in RB; however, no change in their expression was found in KL. Both TCH4 genes might be the key reason for the decrease in FT of the sensitive genotype (RB).

The members of several kinase families are involved in cold signal transduction, including CDPK, CIPK, CRLK, CAMTA, and CNGC. The members of these gene families play an important role in regulating FT. In our study, we identified several DEGs, including 2 CDPK genes, 5 CIPK genes, 1 CAMTA gene, and 4 CNGC genes, indicating that the FT of kiwifruit might be enhanced through the Ca^2+^ signaling pathway. Interestingly, recent research in rice showed that cyclic nucleotide-gated channel (OsCNGC9) positively regulates chilling tolerance by mediating cytoplasmic calcium elevation [65]. In KL, 4 GNGC genes had higher expression than that in RB. These GNGC genes may be candidate genes for explaining the difference in FT between the genotypes.

The MAPK signaling pathway was also significantly induced, showing that this pathway is associated with cold stress responses [66]. Calcium/calmodulin binds to CRLK1 and activates it, after which CRLK1 interacts with MEKK1 (mitogen-activated protein kinase kinase kinase 1), leading to MAPK activation and freezing tolerance. MAPK cascades transduce cold signals into intracellular responses using various classes of protein kinases. With respect to kinases, some genes encoding MPKs (mitogen-activated protein kinases) were shown to be upregulated in response to low temperature in *A. thaliana* [67]. Some researchers identified another MAP3K that played an important role in cold stress signal transduction to enhance FT in *Poncirus trifoliata* [68]. In our study, a transcript (MWXS20249_01mi_transcript_12622) encoding MPK3/6 was identified, and its expression increased under cold stress. Cold-activated MPK3/6 phosphorylates ICE1 proteins to improve their stability and transcriptional activity, which consequently regulates *CBF* expression in and FT of plants.

### Transcriptional regulatory pathways involved in the cold response

TFs, e.g., AP2/ERF, bHLH, and MYB, are differentially expressed at low temperature and have been reported to be associated with FT in *A. thaliana*. Moreover, the members of the AP2/ERF TF family were the most abundant TFs according to our data. The *CBF* gene belongs to the AP2/ERF family; this gene can induce the expression of cold-responsive genes (CORs) that are involved in multiple abiotic stress responses [69]. Previous studies have indicated that AtCBF1, 2, and 3 are responsive to cold stress in *A. thaliana*. Overexpression of the *CBF* gene was shown to activate the expression of COR genes and increase the FT of plants. In our study, the CBF1 gene was identified as a candidate gene for enhancing the FT of KL, and its upstream regulated genes, including *CAMTA*, *EBF*, *EIN*, *ZAT10*, and *ZAT12,* were also induced under cold stress in KL (Table 2). Members of the bHLH family also have a positive function in enhancing FT. bHLH TFs, which act upstream of *CBF*, can bind to the G/E-box motif of the *CBF* gene and subsequently improve FT by activating COR genes [70, 71]. Previous studies have shown that overexpression of *ICE* improves the FT of *A. thaliana*. According to our data, five *MYC2* genes belonging to the bHLH family were upregulated in KL, whereas there were no significant changes in RB (Fig. 9c). MYB transcription factors may play an important role in the low-temperature response and changes in the expression levels of COR genes, affecting the physiological response to resist adverse environmental conditions. For example, *AtMYB15* is a transcription regulatory gene that negatively regulates the FT of *Arabidopsis* [72]. *MYB*s were differentially expressed between KL and RB under cold stress, implying a key role of *MYB* genes in the low-temperature response under cold stress (Fig. 9d). Overall, TFs are crucial mediators in regulating the expression of COR genes to affect the FT of kiwifruit.

### WGCNA of the gene network regulation of the FT mechanism

Freezing tolerance is the result of a complex biochemical pathway that is involved in the regulation of multiple genes; therefore, a single gene is not enough to explain FT. Genes involved in multiple metabolic pathways are usually regulated through coordinated expression, so correlation models are often used to identify gene coexpression networks [73, 74]. KEGG analysis and TF analysis revealed several candidate genes that respond to cold stress in kiwifruit in our study. We obtained 7 gene expression modules via WGCNA, of which one module was strongly correlated with starch-sucrose metabolism. The blue module from the WGCNA correlated significantly with beta-GC, TPS, and BAM. Through hub gene searching in the blue module, *beta-GC*, *TPS5*, *BAM3.1*, *CBF3*, *MYC2*, and *MYB44* were screened as key genes. These results indicated that the above 6 candidate genes were key players in the cold response in kiwifruit. In general, the overview of these genes using coexpression networks would strengthen our understanding of coordinated regulation of multiple genes involved in the FT of kiwifruit.

**Fig. 12:**
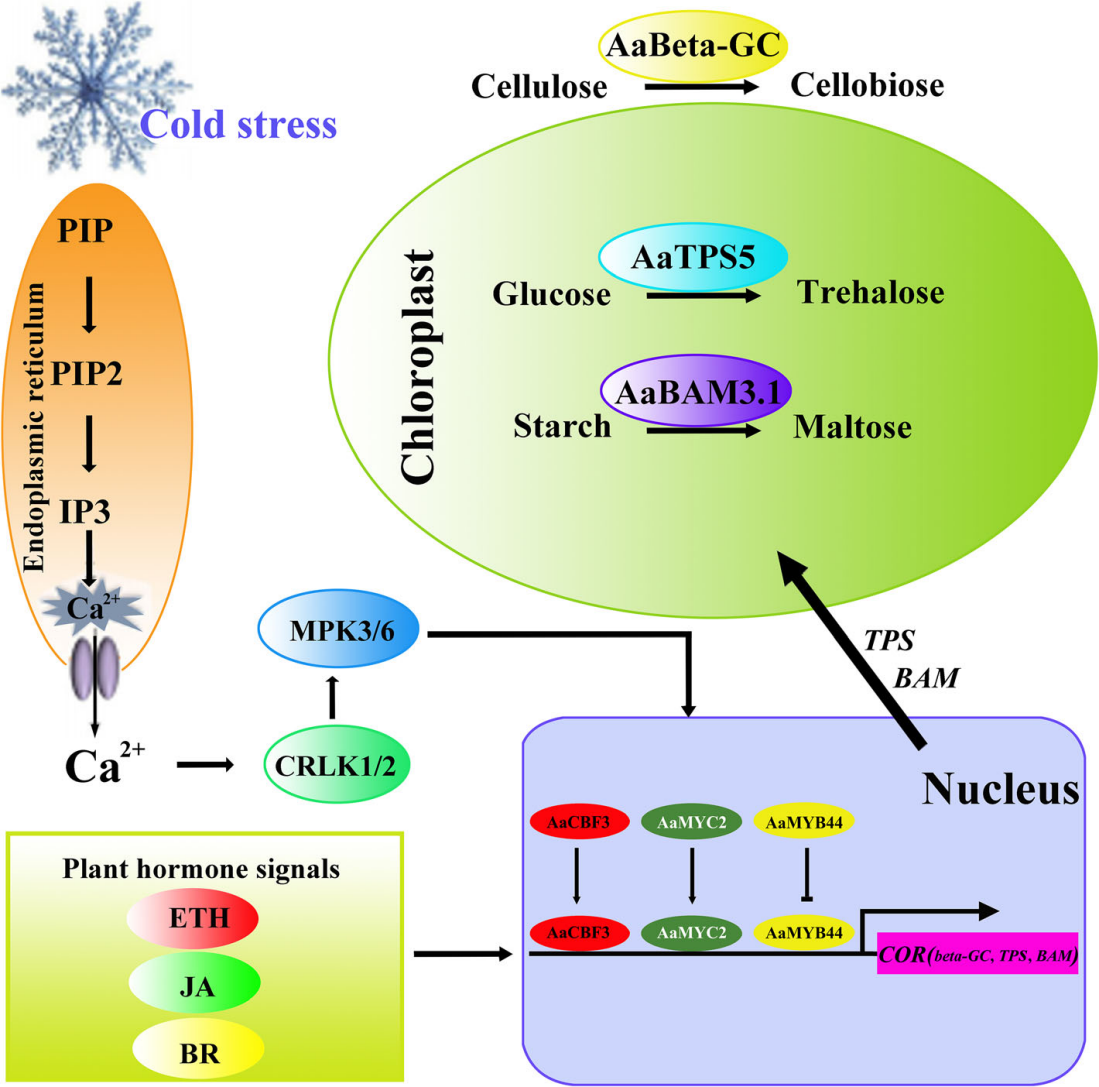
Putative candidate model of gene involvement in the FT of kiwifruit. Phosphatidylinositol phosphate, PIP; phosphatidylinositol bisphosphate, PIP2; inositol 3-phosphate IP3; ethylene, ETH; jasmonic acid, JA; brassinolide, BR

Based on our results, we hypothesized that when kiwifruit are subjected to cold stress, various metabolites act as low-temperature sensors, after which cells release calcium through phosphatidylinositol signaling and inositol phosphate metabolism. Calcium interacts with CRLK to transduce cold signals through the MAPK signaling pathway, and then the MAPK signaling pathway triggers signal transduction via TFs such as ERF, bHLH, and MYB TFs. Moreover, plant hormones including ETH, JA, and BRs also serve as signaling molecules to regulate COR gene expression. Subsequently, the starch-sucrose pathway, which regulates osmolyte and redox balance, and the production of COR genes, including beta-GC, TPS, and beta-amylase, through activation of the CBF pathway and non-CBF pathway, in turn enhance FT. Overall, the accumulation of soluble sugars, trehalose, and maltose plays an important role in resisting adverse temperature conditions in kiwifruit. A diagram explaining the process involving kiwifruit FT is shown in Fig. 12.

Our results indicated that signaling transduction pathway and starch and sucrose pathway were key pathways to regulate kiwifruit freezing tolerance and that key genes involved in these pathways might lead to different FT between freezing tolerant genotype and sensitive genotype. However, one limitation in our study should also be considered: keeping in mind the limited number of samples (two samples) in current study, comparative transcriptome of two genotypes might lead to identify fake DEGs which were not related to freezing adaption but to different genetic background. Therefore, firstly, the two materials were compared with their respective untreatment materials (control) to sort out low temperature induced transcripts when we screened the differential transcripts. Secondly, transcriptome is a feasible method to identify key genes, the qRT-PCR results of key candidate genes validate authentic DEGs. Moreover, it is better method to find key genes through genome-wide association study (GWAS) or bulk-sequencing-analysis (BSA) after the genome of *A. arguta* is sequenced. In future studies, larger and more diverse samples should be investigated to further identify key DEGs. Additionally, the relationship of TFs and 3 sub-pathways including starch degradation, trehalose synthesis and cellulose degradation need to be clearly confirmed. Future studies also need to focus on how certain changes in the hub gene can affect other pathways or traits.

## Conclusions

In the present study, an integrated transcriptome profile of contrasting kiwifruit genotypes under low temperature was analyzed via RNA-seq combined with SMRT and NGS technologies to reveal the FT mechanism. We identified the following genes in response to low temperature: (1) starch and sucrose metabolism-related genes (beta-GC, TPS5, BAM3.1) and (2) transcription factor genes (CBF3, MYC2, MYB44). Additionally, the ‘phosphatidylinositol signaling system’, ‘inositol phosphate metabolism’, ‘MAPK signaling pathway’, ‘plant hormone signal transduction’, and ‘starch and sucrose metabolism’ processes were involved in the cold response. These processes might play an important role in the responses to low temperature in kiwifruit. Our results provide novel viewpoints on a set of molecular mechanisms of kiwifruit FT. In future work, we will concentrate on the functional verification of the structural genes *beta-GC*, which is related to cellulose degradation, *TPS*, which is related to trehalose synthesis, and *BAM*, which is related to starch degradation, and explore how they work in response to cold stress.

## Methods

### Plant materials and cold treatment

Two *A. arguta* (Sieb. et Zucc.) Planch. et Miq. genotypes, ‘Kuilv male’ (KL) and ‘Ruby-3’ (RB), were collected in the national kiwifruit germplasm repository of Zhengzhou Fruit Research Institute, CAAS, Henan Province, China. KL originated from Jilin Province (125°E, 44°N; China), and RB originated from Henan Province (113°E, 34°N; China). Tissue culture-generated seedlings were planted in 3-L pots and then grown for 3 years. One-year-old shoots were collected in early January 2020, and then the detached shoots were put into a freezer in darkness. Three biological replicates of shoots for RNA sequencing (RNA-seq) were collected randomly from all plants of each genotype under − 25 °C treatment at 0 h, 1 h, and 4 h. At the same time, shoots were collected for measurement of physiological indices. The collected samples were frozen immediately in liquid nitrogen and stored in a − 80 °C freezer for further study.

### Histological, physiological and biochemical analyses

The anatomical structure of the shoots was observed by the paraffin wax method for both genotypes [75]. In brief, the shoot samples were immediately submerged in FAA (5% formaldehyde:6% acetic acid:63% alcohol:26% H_2_O) fixative solution for 72 h. The shoot samples were then gradually dehydrated using an alcohol gradient. Later, the shoot sections were embedded in liquid paraffin wax (temperature 56–58 °C). The shoot sections (15 μm) for optical microscopy observations were sliced by a microtome. Each time point was represent by at least five images per sample.

The activities of beta-glucosidase (beta-GC), trehalose-6-phosphate synthase (TPS), and beta-amylase (BAM) and the contents of malondialdehyde (MDA), hydrogen peroxide (H_2_O_2_), soluble sugars, maltose, and trehalose were measured using corresponding assay kits according to the manufacturer’s instructions (Suzhou Comin Bioengineering Co., Ltd., Suzhou, China). The experiments involved three biological replicates.

### RNA extraction and RNA-seq

The samples were subjected to RNA extraction using an RNA Isolation Kit (Waryoung, China). Both the quality and concentration of the RNA samples were subsequently measured using an Agilent 2100 Bioanalyzer (Agilent Technologies, USA), a Qubit 3.0 Fluorometer (Invitrogen, USA), and a NanoDrop 2000 spectrophotometer (NanoDrop Products, USA). For Illumina libraries, the total RNA was subjected to mRNA purification using magnetic beads linked with oligo (dT) beads. The mRNA molecules were fragmented into short fragments, the short fragments were used as templates to synthesize cDNA, and the library was constructed. The library was subjected to 150 bp paired-end sequencing using the NGS tool of the Illumina HiSeq 2500 platform (Illumina Inc., USA). For PacBio libraries, cDNA was prepared using a SMRTer PCR cDNA Synthesis Kit (Clontech, USA), and PacBio libraries were constructed according to the PacBio protocol (http://www.pacb.com/wp-content).

### Data processing

The short-read data were obtained via NGS, and raw reads were filtered by removing adapter reads, poly-N reads and low-quality reads to obtain clean reads [63]. The PacBio sequencing results were analyzed using the SMRT Pipe analysis workflow of the PacBio SMRT Analysis software suite (http://www.pacb.com/products-and-services/analytical-software/smrt-analysis). In brief, to obtain high-quality reads, the SMRT sequencing results were filtered by removing polymerase reads with a length of < 50 bp and a quality of < 0.75. The high-quality reads were subsequently chosen to obtain error-corrected circular consensus sequences (CCSs) for which the full passes were ≥ 0 and the quality was > 0.75. The CCSs were regarded as full-length (FL) transcripts when the 5′ primer sequence, 3′ primer sequence, and a poly-A tail were present. The artificial chimeric sequences were produced by direct linkages of two cDNA template strands due to the low concentrations of adapters or SMRTbell, whereas nonchimeric sequences in the FL sequence were considered to be FL-nonchimeric (FLNC) sequences. The FLNC transcripts were identified by searching for 5′ tail cDNA primers and poly-A tail signals in the CCSs. FLNC transcripts with a high-quality base were obtained for subsequent analysis.

### Functional annotations of transcripts and identification of DEGs

Due to the absence of a reference genome for tetraploid kiwifruit, a reference transcriptome was assembled de novo from SMRT transcript data. Unigene sequences were annotated in various databases, and annotation information of the unigenes was obtained for those with an E-values of at least 1e-5. Five databases were used: the nonredundant protein sequence (NR) database, Gene Ontology (GO) database, Eukaryotic Ortholog Groups (KOG) database, Homologous Protein Family (Pfam) database, and Kyoto Encyclopedia of Genes and Genomes (KEGG) database. iTAK was used to predict plant transcription factors (TFs), and the TF family classification used by the database was adopted to identify TFs by hmmscan. The DESeq tool (R package version 1.10.1) was used to identify the DEGs (differentially expressed genes) between different comparison groups, and filtered standards were adjusted according to *P*-value < 0.05 and |log2 (fold-change)| ≥1. Via KEGG and GO databases, the enriched DEGs were used to identify significant metabolic pathways, and the filtered standard was an FDR-adjusted *P*-value of < 0.05.

### Validation and expression analysis of DEGs by quantitative reverse transcription-PCR (qRT-PCR)

First-strand cDNA was synthesized from a total of 2 μg of template RNA using a ReverTra Ace qPCR RT Kit (Toyobo, Osaka, Japan) according to the manufacturer’s instructions. qRT-PCR was subsequently performed on a LightCycler 480 real-time PCR system (Roche, Basel, Switzerland) using 2x SYBR Green I Master Mix (Asbio Technology, Inc.) and the first-strand cDNA template. The qRT-PCR protocol was as follows: 95 °C for 5 min, followed by 45 cycles of denaturation at 95 °C for 10 s, annealing at 60 °C for 20 s, and extension at 72 °C for 20 s. The relative expression levels of each sample were calculated in relation to the expression of the reference gene *β-actin* using the 2^-∆∆CT^ method. Three independent biological replications were included. All the primers used in this study are shown in Table S5.

### Gene coexpression network analysis and network visualization

Weighted gene coexpression network analysis (WGCNA) was performed using the WGCNA package (R package version 1.69) with the default parameters. Using the FPKM values of the DEGs, an adjacency matrix was constructed. The resulting adjacency matrix was then converted into a topological overlap matrix (TOM). A dynamic hybrid tree-cut algorithm (via the R package dynamicTreeCut, v.1.63–1) was used to detect modules, with the default settings. The phenotypic data were used to identify the related modules, and a correlation matrix between phenotypes and gene modules was constructed using the WGCNA package. The genes from the related module were visualized using Cytoscape (v.3.8.2).

### Statistical analyses

Two-way analysis of variance (ANOVA) accompanying Tukey’s test was performed for all data using SPSS 22 (IBM, Inc., Armonk, NY, USA) and Prism 7 (GraphPad Software, Inc., La Jolla, CA, USA). Each experiment was repeated at least three times independently, and Pearson’s one-tailed test was conducted at the *P* < 0.05 significance level.

## Supplementary Information

**Additional file 1 Supplementary Fig. 1.** GO functional classifications of all the unigenes.

**Additional file 2 Supplementary Fig. 2.** KEGG functional classifications of all the unigenes.

**Additional file 3 Supplementary Fig. 3.** Phylogenetic tree of candidate genes and homologous genes. (a) *Beta-GC*, (b) *TPS5*, (c) *BAM3.1*, (d) *CBF3*, (e) *MYC2*, and (f) *MYB44*.

**Additional file 4 Table S1.** DEGs related to Starch and sucrose metabolism, MAPK signaling pathway, Phosphatidylinositol signaling system, Inositol phosphate metabolism, Arginine and proline metabolism.

**Additional file 5 Table S2.** DEGs related to hormone and calcium signal.

**Additional file 6 Table S3.** DEGs related to TFs.

**Additional file 7 Table S4.** Top 100 hub genes in the blue module.

**Additional file 8 Table S5.** Primers used for quantitative real-time RT-PCR analysis.

## Data Availability

The raw data generated in this study have been deposited in the NCBI Short Read Archive (SRA) under the accession number PRJNA681641. All other relevant data contained within the paper are available in the paper and Supplementary Files.
