# Supplementary figures and images for: Full-length transcriptome profiling reveals insight into the cold response of two kiwifruit genotypes (A. arguta) with contrasting freezing tolerances
Source: BMC Plant Biol. 2021 Aug 11;21:365. doi: 10.1186/s12870-021-03152-w (PMC8356467; doi:10.1186/s12870-021-03152-w)

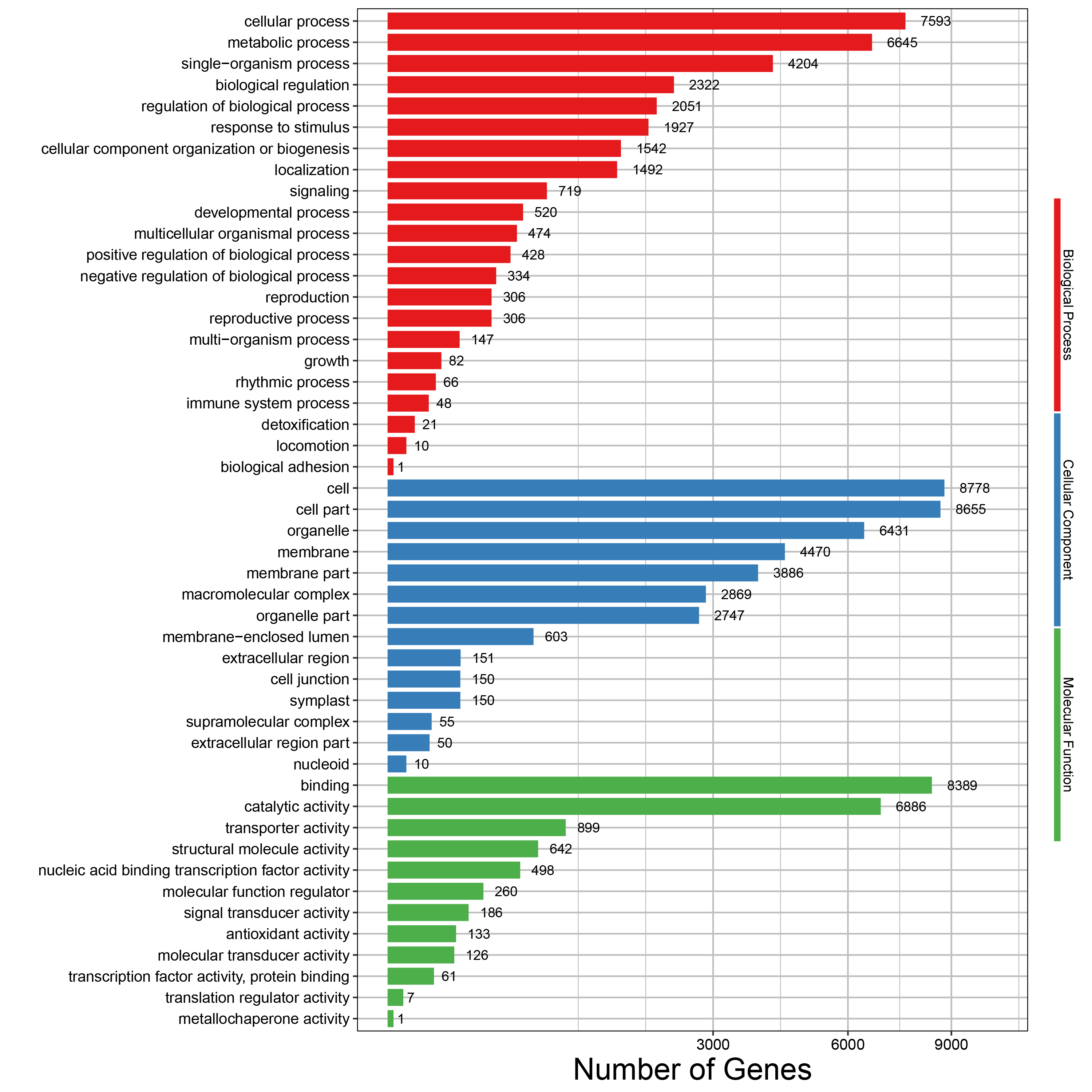

Supplement: Supplementary file 1 — Additional file 1 Supplementary Fig. 1. GO functional classifications of all the unigenes. [file 12870_2021_3152_MOESM1_ESM.jpg]

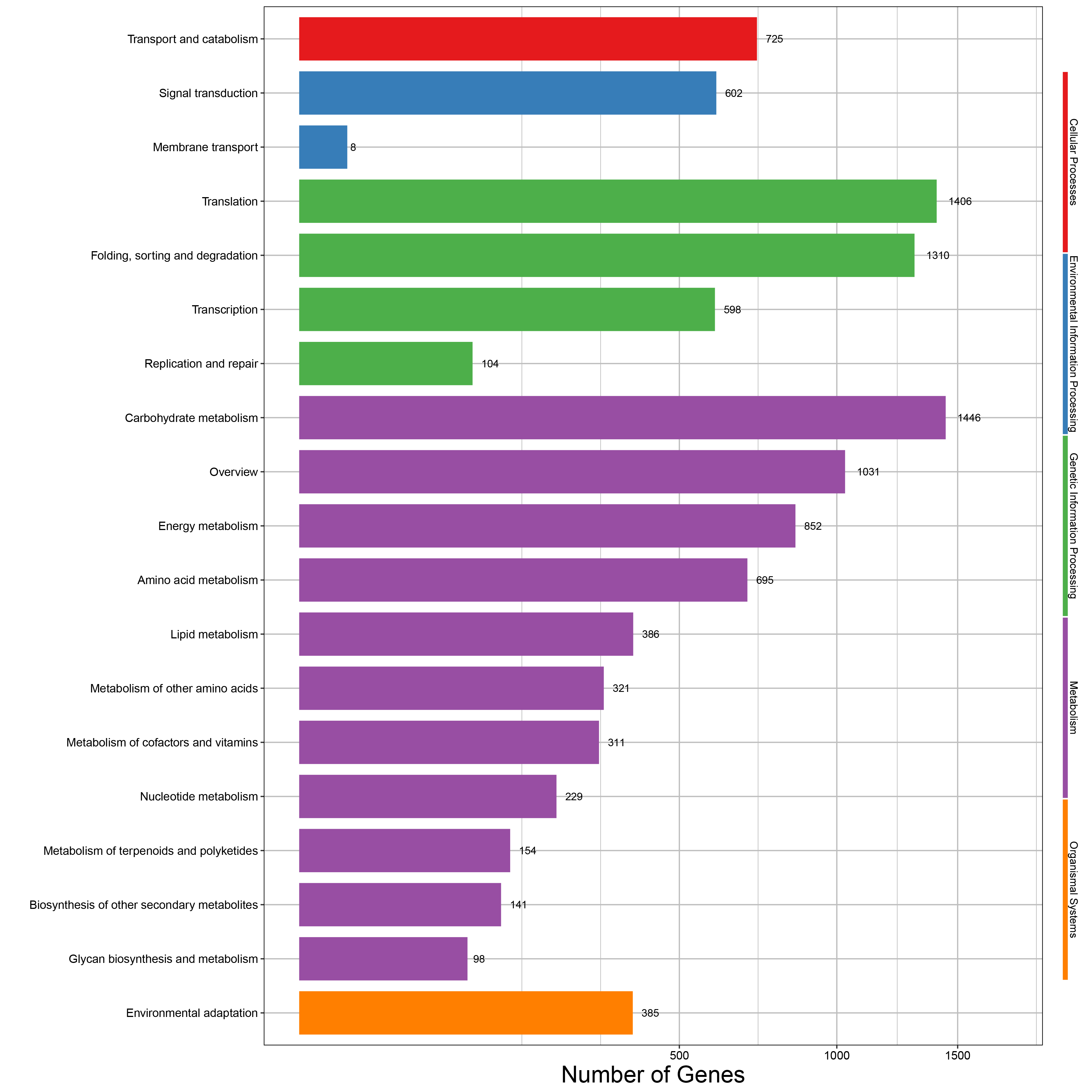

Supplement: Supplementary file 2 — Additional file 2 Supplementary Fig. 2. KEGG functional classifications of all the unigenes. [file 12870_2021_3152_MOESM2_ESM.jpg]

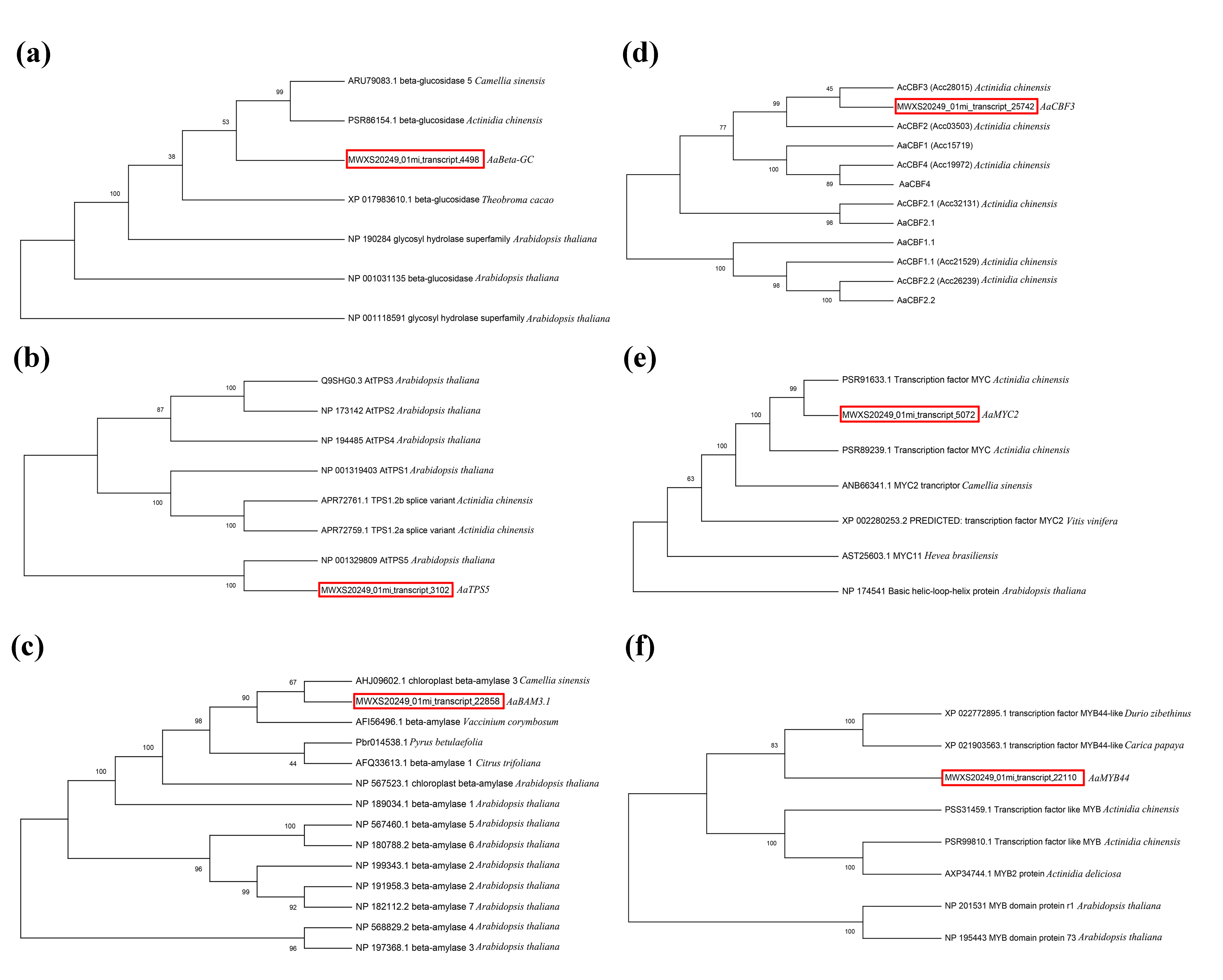

Supplement: Supplementary file 3 — Additional file 3 Supplementary Fig. 3. Phylogenetic tree of candidate genes and homologous genes. (a) Beta-GC, (b) TPS5, (c) BAM3.1, (d) CBF3, (e) MYC2, and (f) MYB44. [file 12870_2021_3152_MOESM3_ESM.jpg]
