# Supplementary material for: Full-length transcriptome profiling reveals insight into the cold response of two kiwifruit genotypes (A. arguta) with contrasting freezing tolerances
Source: BMC Plant Biol. 2021 Aug 11;21:365. doi: 10.1186/s12870-021-03152-w (PMC8356467; doi:10.1186/s12870-021-03152-w)
Supplement: Supplementary file 8 — Additional file 8 Table S5. Primers used for quantitative real-time RT-PCR analysis. [file 12870_2021_3152_MOESM8_ESM.docx]

**Table S5 Primers used for quantitative real-time RT-PCR analysis**

| Gene name | **Forward (**5’-3’**)** | **Reverse (**5’-3’**)** |
| --- | --- | --- |
| *Sucrose synthase* | TCCGCTCACCGCAACGAACT | CAAATGGAGGCAGAACTATG |
| *CBH1* | TTTTCCCCCATACCCTCTCG | ATCCTGCACGTGTTGGTGAT |
| *Beta-GC* | TGACCCCATCGTTGCCATCT | AATGTTGGGCAGGTTGAGCG |
| *Hexokinase* | TGGACCTCGGTGGCACAAAC | CAACATCTTGTCCAACCGCA |
| *TSP5* | CGTCGGGTGCTTGAAGGCGG | TAGGCTTGCCACAGAGACCG |
| *ADP-glucose pyrophosphorylase* | GGACGCCAAGAACAAAAACG | CTCGGCTGTTGTCCATAGGG |
| *SS* | TGGGCACCGAGTTATGACAG | TTTTCCCCCATACCCTCTCG |
| *SBE* | CGTCAATGGGACTTGGTGGA | CTCCCAAGCATCACTGTCCA |
| *BAM3.1* | ACCTACCAATAGCGCGGATG | AACTAACCCTTCTGGCGAGC |
| *CBF3* | CAGATTGCACGCATTCCGAG | CGCAAACCCACTTGTCTGTG |
| *MYC2* | ATTTGTGGACGGACGATAAC | TTGTAGTACCCGTCGCCCCA |
| *MYB44* | GGTCCGTGGAGTCCTGAAGA | GGTCCGTGGAGTCCTGAAGA |
| *AaActin* | TGCATGAGCGATCAAGTTTCAAG | TGTCCCATGTCTGGTTGATGACT |
